# State of the Art Review on Genetics and Precision Medicine in Arrhythmogenic Cardiomyopathy

**DOI:** 10.3390/ijms21186615

**Published:** 2020-09-10

**Authors:** Viraj Patel, Babken Asatryan, Bhurint Siripanthong, Patricia B. Munroe, Anjali Tiku-Owens, Luis R. Lopes, Mohammed Y. Khanji, Alexandros Protonotarios, Pasquale Santangeli, Daniele Muser, Francis E. Marchlinski, Peter A. Brady, C. Anwar A. Chahal

**Affiliations:** 1 Department of Cardiology, Royal Papworth Hospital, Cambridge CB2 0AY, UK; viraj.patel@nhs.net; 2 Department of Cardiology, Inselspital, Bern University Hospital, University of Bern, 3010 Bern, Switzerland; babken.asatryan@insel.ch; 3 School of Clinical Medicine, University of Cambridge, Cambridge CB2 0SP, UK; bs585@cam.ac.uk; 4 Clinical Pharmacology, William Harvey Research Institute, Barts and The London School of Medicine and Dentistry, Queen Mary University of London, London EC1M 6BQ, UK; p.b.munroe@qmul.ac.uk; 5 NIHR Barts Cardiovascular Biomedical Research Centre, Barts and The London School of Medicine and Dentistry, Queen Mary University of London, London EC1M 6BQ, UK; 6 Division of Cardiovascular Medicine, Hospital of the University of Pennsylvania, Philadelphia, PA 19104, USA; Anjali.owens@pennmedicine.upenn.edu (A.T.-O.); pasquale.santangeli@pennmedicine.upenn.edu (P.S.); daniele.muser@gmail.com (D.M.); francis.marchlinski@pennmedicine.upenn.edu (F.E.M.); 7 Department of Cardiology, St Bartholomew’s Hospital, London EC1A 7BE, UK; luis.lopes1@nhs.net (L.R.L.); m.khanji@qmul.ac.uk (M.Y.K.); alexanderproton@gmail.com (A.P.); 8 Centre for Heart Muscle Disease, UCL Institute of Cardiovascular Science, London WC1E 6BT, UK; 9 Department of Cardiovascular Medicine, Mayo Clinic, Rochester, MN 55905, USA; pab11@me.com; 10 Division of Cardiology, Department of Medicine, Advocate Illinois Masonic Medical Center, Chicago, IL 60657, USA

**Keywords:** arrhythmogenic cardiomyopathy, genetics, arrhythmogenic right ventricular cardiomyopathy, desmosome, cardiac arrhythmia, sudden cardiac death, genotype phenotype correlation

## Abstract

Arrhythmogenic cardiomyopathy (ACM) is an inherited cardiomyopathy characterised by ventricular arrhythmia and an increased risk of sudden cardiac death (SCD). Numerous genetic determinants and phenotypic manifestations have been discovered in ACM, posing a significant clinical challenge. Further to this, wider evaluation of family members has revealed incomplete penetrance and variable expressivity in ACM, suggesting a complex genotype-phenotype relationship. This review details the genetic basis of ACM with specific genotype-phenotype associations, providing the reader with a nuanced perspective of this condition; whilst also proposing a future roadmap to delivering precision medicine-based management in ACM.

## 1. Introduction to Arrhythmogenic Cardiomyopathies: Evolving Concepts

Arrhythmogenic cardiomyopathy (ACM) is a relatively new term, used to describe a phenotypically and genetically heterogenous myocardial disease characterised by high ventricular arrhythmia burden, myocardial fibrosis/scarring, and an increased risk of sudden cardiac death (SCD) [1]. Over the last two decades, comprehensive evaluation of ACM patient cohorts has exposed a complex genetic background. Furthermore, extending clinical evaluation to proband’s family members has revealed incomplete penetrance and variable expressivity in ACM, and specific phenotypic manifestations linked to distinct genetic forms [2]. While the pathophysiological mechanisms involved in ACM are largely unclear (with multiple theories and potential contributors proposed), applying genetic knowledge to clinical practice has fundamentally changed clinical care; from a generalised patient-only approach to a targeted family approach, often with integration of novel, family specific knowledge.

In this review, we provide the reader with an overview of what is known about the genetic basis of ACM and associated phenotypes. We highlight common challenges in the field of ACM genetics and propose future directions that may help to further refine our understanding and clinical management of ACM.

## 2. Classification of Arrhythmogenic Cardiomyopathies

The evolving terminology used to describe different forms of “arrhythmogenic cardiomyopathies” reflect the different phases of knowledge surrounding this disease over the last four decades. Early descriptions of ACM led to the presumed pathophysiological model of a developmental defect of the right ventricle (RV), hence, was given the term ‘Arrhythmogenic Right Ventricular Dysplasia’ (ARVD) [3]. Subsequent investigation revealed patients had normal hearts at birth and disease was progressive and genetically determined, leading to the reclassification as a cardiomyopathy and the name, ‘Arrhythmogenic Right Ventricular Cardiomyopathy’ (ARVC) [4]. However, to this date, both terms ARVC and ARVD are often used indiscriminately.

**Figure 1:**
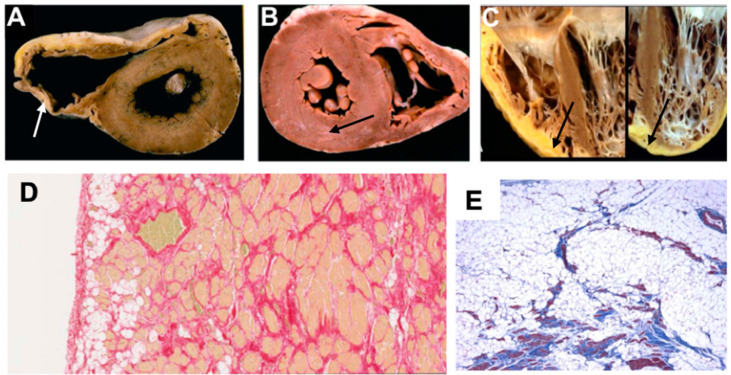
Explanted heart images showing pathological features of different phenotypes in ACM, adapted from Calkins (2013) [10], Thiene et al. (2016) [11], Corrado et al. (2020) [12]. (**A**) Gross specimen shows dilated thin RV, fatty replacement of entire RV free wall epicardium and thin fibrotic endocardium (white arrow). (**B**) Gross specimen shows little evidence of RV involvement, however subepicardial grey band of fibrotic tissue (black arrow) is seen in the posterolateral section of the LV. (**C**) Gross specimen shows fibro-fatty biventricular involvement (black arrows). (**D**) Histology image shows fat replacement extending from epicardium to endocardium. (**E**) Histology image with trichrome staining identifies fibrous scars within fat tissue. LV = left ventricle; RV = right ventricle.

ARVC has long been used to describe a disease confined to the RV with little or no left ventricle (LV) impairment [5], which is described pathologically by fibrofatty replacement of the right ventricular myocardium in advanced stages. However, LV involvement, described in the minority of cases even back in the 18th century by Lancisi, has been recently recognised to be rather common in the so-called “ARVC” hearts. Moreover, cases with more pronounced dominant left-ventricular involvement, referred to as arrhythmogenic left-ventricular cardiomyopathy (ALVC) (or left-dominant arrhythmogenic cardiomyopathy), have also been described [6,7] (Figure 1). Biventricular involvement occurs in 60% of ARVC diagnoses [8]; literature contains the terms diffuse biventricular fibrosis and/or fibrofatty replacement referring to biventricular arrhythmogenic cardiomyopathy (BivACM) characterised in both pathology reports and imaging studies [7,9]. In parallel, genetic forms of dilated cardiomyopathy (DCM) with subepicardial fibrosis and high ventricular arrhythmia burden starting at early disease stages—a feature more characteristic to ARVC/ALVC—were discovered, thereby largely contributing to the need for a new definition and classification of these cardiomyopathies. For the interested reader, we summarise the different nosology in chronological order in Table 1.

The term “ACM” has been used in the 2019 Heart Rhythm Society (HRS) expert consensus statement on evaluation, risk stratification and management of ACM to reflect the expanding spectrum of conditions of either genetic or non-genetic aetiology involving the RV, the LV or both ventricles, whose typical feature is prominent non-ischaemic ventricular myocardial fibrosis/scarring and ventricular arrhythmias [1,12]. The rationale for using this ‘umbrella term’ is conditions manifesting with such an ACM phenotype are associated with a particularly high risk of SCD since myocardial fibrosis acts as a substrate for malignant ventricular arrhythmias [12]. Accordingly, the insertion of an implantable cardiac defibrillator (ICD) in ACM patients for primary prevention should be considered in the presence of large arrhythmogenic ventricular scarring, regardless if systolic ventricular function is not severely reduced [1]. The HRS definition is broad and includes Chagas cardiomyopathy, hypertrophic cardiomyopathy (HCM) and cardiac amyloidosis, the latter of which is in itself a broad range of diseases with distinct phenotypes, none of which are the focus of this review.

**Figure 2:**
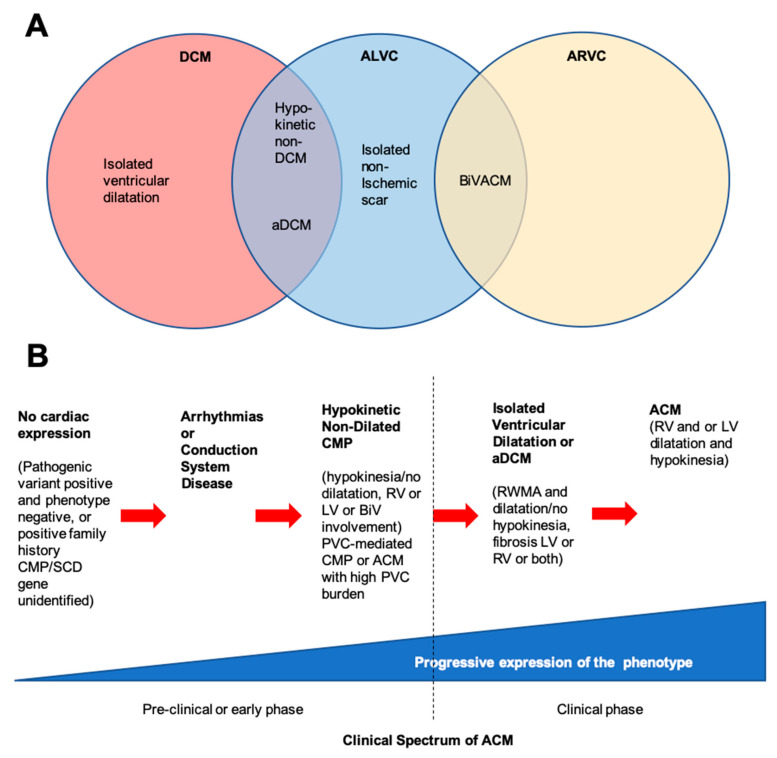
Spectrum of clinical presentations of right ventricular disease, left ventricular disease, and dilated cardiomyopathy. (**A**) ACM and ALVC show overlapping phenotypes with ARVC, but the pathology is seen primarily in the left ventricle (although right ventricle is also usually affected). (**B**) Timeline showing the clinical features of ACM. Hypokinetic non-dilated cardiomyopathy and isolated-ischaemic scar pertain to the pathology where there is marked ventricular scarring as identified by cardiac MRI. Modified from Elliott et al. (2019) [22] and Pinto et al. (2016) [24]. ACM = arrhythmogenic cardiomyopathy; aDCM = arrhythmogenic dilated cardiomyopathy; ALVC = arrhythmogenic left ventricular cardiomyopathy; ARVC = arrhythmogenic right ventricular cardiomyopathy; BiV = biventricular; BivACM = biventricular arrhythmogenic cardiomyopathy; CMP = cardiomyopathy; DCM = dilated cardiomyopathy; LV = left ventricle; PVC = premature ventricular contraction; RV = right ventricle; RWMA = regional wall motion abnormality; SCD = sudden cardiac death.

In 2017, an international group of experts held a roundtable discussion to define ACM, which included classical ARVC, ALVC, biventricular ACM but also recognised a distinction in some of the classical non-ischaemic dilated cardiomyopathies with a propensity for arrhythmias (including bradycardias with conduction system disease, premature ventricular complexes (PVC), ventricular tachyarrhythmias (VTA)), where this may be the dominant feature with mild or no observable structural abnormalities referred to as arrhythmogenic DCM (aDCM) [22]. It is also increasingly recognised that some will have mild hypokinesia, and non-dilated left ventricle, either as a very early stage of the disease or a forme fruste for classical DCM [24]. Myocardial fibrosis leading to re-entrant arrhythmias are the most common form of sustained ventricular arrhythmias. However, focal automaticity is also possible, thought to be caused by additional mechanisms, such as calcium dysregulation, but represent an important contribution to disease outcome. The distinction between ALVC and aDCM in practical terms may be challenging, particularly in cases, which have mild phenotypic expression, or are increasingly detected early in the disease process due to better cascade family screening (Figure 2).

To further refine the spectrum of diseases considered as ACM, an International Expert Panel has recently proposed the Padua criteria for the diagnosis of ACM [23]. These criteria are aimed to upgrade the 2010 revised Task Force criteria (TFC) for the diagnosis of ARVC and introduce new diagnostic criteria regarding tissue characterisation findings by contrast-enhanced cardiovascular magnetic resonance (CMR) imaging, depolarisation/repolarisation ECG abnormalities, and ventricular arrhythmia features for diagnosis of ALVC. While the Padua criteria are very new and require validation in clinical studies, they provide a clear framework for diagnosis of ACM, rather than the HRS umbrella term. These criteria also acknowledge there may be subtle regional or global abnormalities of contractile function without dilatation of the LV or RV [25,26].

**Figure 3:**
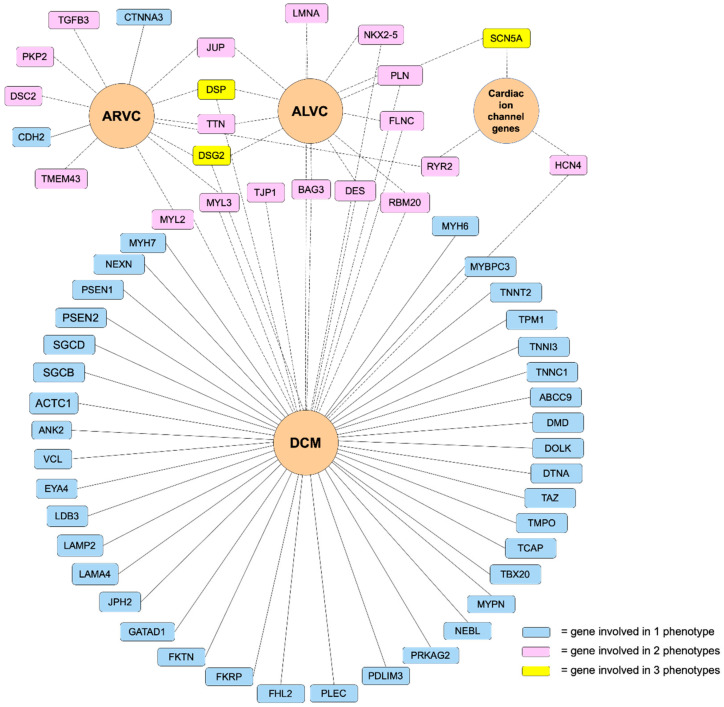
Genetic mutations associated with arrhythmogenic cardiomyopathy. Overlapping genetic determinant implicated in ACM identified from our clinical experience. ACM = arrhythmogenic cardiomyopathy; ALVC = arrhythmogenic left ventricular cardiomyopathy; ARVC = arrhythmogenic right ventricular cardiomyopathy; DCM = dilated cardiomyopathy.

Defining the ACM phenotype and the genes involved is challenging, this is reflected in Figure 3, which demonstrates ACM’s genetic heterogeneity and genetic pleiotropy. We will focus on the desmosomal and extra-desmosomal genes, which cause the classical ARVC phenotype, the biventricular phenotype and the ALVC/aDCM phenotype.

## 3. Genetic Basis of ACM

The discovery of two Mendelian forms of ACM was key to unravelling its genetic basis. Protonotarios et al. (1986) reported a series of families with patients presenting with a combination of cardiomyopathy, palmoplantar keratosis and woolly hair and an autosomal recessive inheritance pattern [27]. All index patients descended from four families residing on the Greek island of Naxos, taking the eponymous name Naxos disease. In 2000, genetic analysis of affected patients revealed a homozygous, truncating two base deletion in the *JUP* gene encoding for plakoglobin (a desmosomal protein) [28]. Similarly, Carvajal-Huerta (1998) reported a group of patients in Ecuador with a familial cardiocutaneous syndrome also inherited in an autosomal recessive fashion [29]. Carvajal syndrome, as it was later named is characterised by ACM (ALVC form), striate palmoplantar keratoderma and woolly hair. Later in 2000, genetic analysis revealed a single homozygous deletion in the *DSP* gene leading to a truncated desmoplakin protein—the most abundant of the desmosome proteins [30]. These early reports prompted targeted genetic analysis of other desmosomal proteins in ARVC patients, which in turn led to identification of pathogenic variants in all known desmosomal genes. More recently, non-desmosomal pathogenic variants have also been identified in ARVC, and many have been implicated in ALVC or biventricular disease forms.

Below, we discuss genes considered to be causative or associated with all three phenotypic subtypes of ACM, presented as a historical narrative; also, we describe distinct phenotypes and major validatory evidence supporting pathogenicity in ACM. The broad classification is desmosomal and non-desmosomal genes. This is summarised in Table 2. Further, we have highlighted exemplar phenotypes for each major gene implicated in ACM (Figure 4, Figure 5, Figure 6, Figure 7, Figure 8, Figure 9, Figure 10, Figure 11, Figure 12, Figure 13, Figure 14, Figure 15, Figure 16, Figure 17, Figure 18 and Figure 19).

### 3.1. Desmosomal Genes

**Figure 20:**
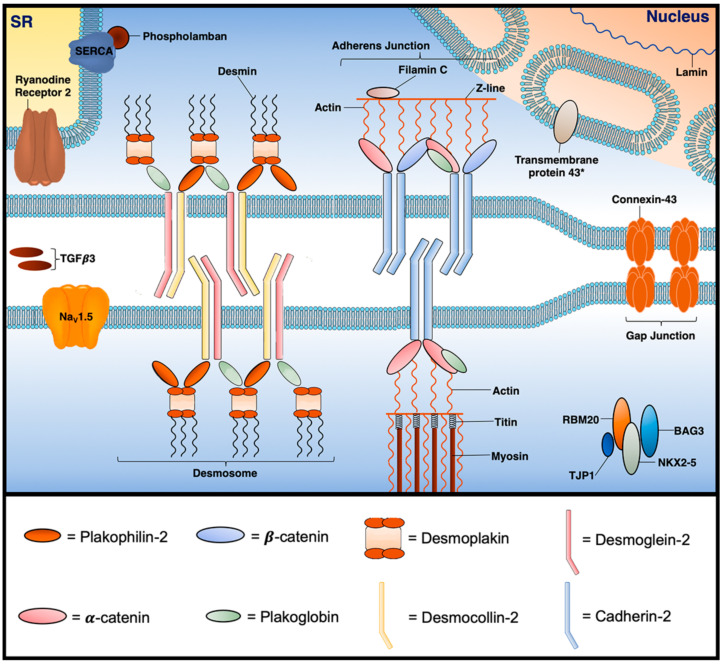
Select proteins implicated in the pathogenesis of ACM at the cardiomyocyte junction. BAG3 = BAG family molecular chaperone regulator 3; Na_v_1.5 = voltage-gated Na^+^ channel 1.5; NKX2-5 = NK2 homeobox 5; RBM20 = RNA-binding motif protein 20; SERCA = sarcoplasmic reticulum Ca^2+^ ATPase; SR = sarcoplasmic reticulum; TGF-𝛽3 = transforming growth factor 𝛽3; TJP1 = tight junction protein. * = location is still unclear.

Desmosomes are calcium-dependent multi-protein junctional anchors described in lay terms as ‘cellular glue’. Whilst desmosomes establish tight extracellular adhesion between neighbouring cells, they also link to the intermediate filament cytoskeleton enabling structural resilience particularly under high mechanical stress. Desmosomal proteins have a number of additional functional roles; most notably, they regulate the transcription of genes involved in adipogenesis and apoptosis, and play a major role in myocardial electrical conduction through regulation of gap junctions and calcium homeostasis. These functions are poorly understood, and specific observations are noted below (a more extensive guide can be found in reference [50]) [51,52]. Desmosomes are part of a larger structure known as the intercalated disc (ID) (Figure 20). Alongside the desmosome, the ID contains several macromolecular complexes with distinct properties: the adherens junction, the gap junction and ion channel. Contrary to previous thought, growing evidence suggests these individual components (in particular, desmosomes, gap junctions and voltage gated sodium channels) interact together to electrically, metabolically and structurally couple neighbouring cardiomyocytes as a single functional unit known as the connexome [52,53].

Desmosomal proteins can be divided into three families known as 1. cadherins; 2. armadillos; and 3. plakins. Desmosomal cadherins (Desmocollin-2 (DSC2) and Desmoglein-2 (DSG2)) are transmembrane proteins that extracellularly bind with adjoining desmosomal cadherins and intracellularly bind to armadillos. When DSC2 and DSG2 are bound, the desmosome adopts a calcium-dependent hyper-adhesive state, which has the ability to shift to a lower affinity state during wound healing and embryonic development [50,51]. The cardiac armadillo family (Plakophilin-2 (*PKP2*) and Plakoglobin (PKG)) is characterised by the armadillo arm repeat-a repetitive tandem amino acid sequence of about 40 residues in length composed of a pair of alpha helices that form a hairpin structure [51]. The armadillos help to stabilise the intracellular desmosomal plaque—they link cadherins to desmoplakin and mediate the interaction between desmoplakin and the intermediate filament, desmin [51]. The desmosomal plakin family consists only of desmoplakin (DSP) which binds the desmosome to the intermediate filament, desmin [51].

Nearly 60% of ARVC patients have a genetic alteration in at least one of the five genes encoding cardiac desmosome proteins: *DSC2, DSG2, PKP, JUP, DSP* [23]. All desmosomal genes have multiple lines of evidence replicated in time to confirm their pathogenicity in ARVC, with many reports providing clinical, histological and functional evidence for causality in the full spectrum of ACM. Desmosomal ARVC predominantly follows autosomal dominant inheritance with incomplete penetrance; exceptions include Naxos disease (triad of autosomal recessive ARVC, palmoplantar keratoderma, and woolly hair) [28,54], and Carvajal syndrome (a variant of Naxos disease with ALVC, associated with early morbidity), caused by recessive mutations in *JUP* and *DSP*, respectively. Around 75% of genotype-positive ARVC cases in American cohorts, and nearly 60% of genotype-positive index cases in European cohorts are caused by single pathogenic variants in *PKP2*, making it the most commonly involved ARVC gene [55,56,57]. Mutations in *DSP* are often associated with ALVC, and *DSG2* and *PKP2* with BivACM, although the latter is observed in all gene groups at later stages of disease progression [7].

In general, autosomal recessive disease tends to have a more advanced phenotype and penetrance, and whilst intuitive to expect adverse outcomes, little data exist to support this [58,59]. In Naxos, disease outcomes were similar to autosomal dominant *PKP2* patients [60]. Desmosomal pathogenic variants usually predispose to younger disease onset and affected patients often have a strong family history of ARVC/SCD [61]. In general, patients with ALVC or biventricular disease are known to have worse outcomes than those with spared LV, and SCD prior to advanced ALVC disease appears to be common [62]. Multiple pathogenic variants have been associated with higher risk of SCD [62].

### 3.2. Non-Desmosomal Genes

Non-desmosomal pathogenic variants are causative of ACM in a significant proportion of cases. While ARVC is more often caused by desmosomal variants, genetic defects in non-desmosomal genes are thought to be more frequently involved in ALVC.

#### 3.2.1. Titin (TTN) (Encoded by *TTN*)

Titin is the largest natural protein (38,138 residues, MR 4200 kDa) and is a giant protein with a length > 1 μm [63,64]. Although the *TTN* gene is not the largest, it is one of the largest with the greatest number of exons (363), as well as the largest single exon (17,106 bp). Titin protein is the third most abundant protein in muscle after actin and myosin, and is an essential component of the sarcomere linking myosin and the Z disc, providing structural support, flexibility, and stability. Different isoforms exist in different muscles, including cardiac muscle. Additional functions include a spring-like region allowing muscle to stretch, as well as chemical signalling and assisting in the formation of new sarcomeres [65].

Titin truncating variants are now the most frequent genetic cause of DCM [66], as well as peripartum cardiomyopathy [67]. Given *TTN*’s size, it is likely to result in greater de novo variant (s) from background mutagenesis. Considering the relatively high frequency of missense variants in the general population, and reclassification of pathogenic to benign classification, currently only truncating (nonsense) and frameshift variants are considered likely to be pathogenic, with a few exceptions.

In ARVC cases with negative desmosomal genetic testing, missense and truncating variants in *TTN* have been reported [40]. In one study of 38 families with TFC positive ARVC, 312 exons of *TTN* expressed in human cardiac titin and entire 3′ UTR were sequenced, identifying 8 unique *TTN* variants in 7 (18%) families, which were absent in 400 ethnically matched controls and genomic databases. One of the pathogenic variants, Thr2896Ile, showed complete segregation with the ARVC phenotype in one large family and mapped within a highly conserved immunoglobulin-like fold (Ig10 domain) located in the spring-like region of titin. The phenotype of *TTN* pathogenic variant carriers was characterised by a history of SCD (5 of 7 families), worsening myocardial dysfunction causing death or cardiac transplantation (8 of 14 cases), frequent cardiac conduction disease (11 of 14), and incomplete penetrance (86%). A subsequent genotype-phenotype natural history study from the same group, compared *TTN* variant carriers to desmosomal carriers and ARVC phenotype-positive but negative for *TTN* or desmosomal pathogenic variants (noncarriers) and noted, *TTN* carriers were more likely to develop conduction system disease and supraventricular arrhythmias than non-carriers [68]. On echocardiography, *TTN* carriers were more likely to have left atrial enlargement, mitral regurgitation (MR) and RV dilation than both desmosomal and noncarriers. However, prognosis (both mortality and morbidity) was worse and T wave inversion (TWI) was more frequent in desmosomal variant carriers.

#### 3.2.2. Lamin A/C (LMNA) (Encoded by *LMNA*)

Lamins are nuclear intermediate filament proteins that support and determine the shape of the nuclear envelope [63,64]. *LMNA* encodes two products—Lamin A and Lamin C via alternative splicing. *LMNA* pathogenic variants have been associated with a broad range of cardiac phenotypes, including cardiac conduction disease, atrial and ventricular arrhythmias, and DCM; as well as extracardiac manifestation such as lipodystrophies, skeletal myopathies, and premature ageing syndromes [41]. *LMNA* was first implicated in ACM by Quarta et al. (2012); 4 out of 108 ARVC patients who tested negative for desmosomal pathogenic variants were found to carry *LMNA* variants [41]. Two of these patients had fibrofatty myocardial changes consistent with ARVC, and staining revealed reduced plakoglobin in the ID—a characteristic finding [41]. However, *LMNA* variants are most commonly associated with an aDCM phenotype with conduction system disease and a high premature ventricular contraction (PVC)/ventricular tachycardia (VT) burden, often being the initial manifestations of laminopathies [69,70,71].

LMNA is a ubiquitously expressed nuclear envelope protein that interacts with chromatin through lamin-associated domains to regulate gene expression [72,73]. In human cardiomyocytes, LMNA interacts with around 20% of the genome and has suppressive effects on expression of several thousand genes [74]. Consequently, pathogenic variants in the *LMNA* gene may suppress expression of genes implicated in inherited arrhythmias, such as *SCN5A* [75]. Although the molecular basis of aDCM and ACM associated with the *LMNA* pathogenic variants have yet to be delineated, the regulatory role of *LMNA* on many genes probably explains the phenotypic pleiotropy of *LMNA*-mediated disease.

The high incidence of SCD in these families had led to recommendations to consider prophylactic implantable cardioverter defibrillators (ICD) for SCD prevention. The 2019 HRS ACM document provides two class IIa recommendations for a primary prevention ICD in *LMNA* pathogenic variant carriers: 1. those with two or more of the following: left ventricular ejection fraction (LVEF) less than 45%, male, non-sustained ventricular tachycardia (NSVT). 2. Those who have an indication for pacing [1]. Reports from large cohorts are still needed to support this. In addition, a recent group have created a *LMNA*-risk VTA calculator using five parameters (sex, non-missense mutation present, type and presence of atrioventricular block, NSVT present, LVEF) to provide a risk prediction score for life-threatening VTA at five years (https://lmna-risk-vta.fr/) [76].

#### 3.2.3. Desmin (DES) (Encoded by *DES*)

Desmin is a muscle-specific cytoplasmic intermediate filament protein that links the Z-disc to the junctional and nuclear membrane [63]. As well as providing structural integrity to cardiomyocytes, DES interacts with other organelles including mitochondria, and is involved in processes that include nucleus position and sarcomere synthesis, sarcoplasmic reticulum and the T-tubular system [77,78]. Mutations in the *DES* gene have been reported with all types of morphological cardiomyopathies and skeletal myopathies. Both autosomal dominant and recessive inheritance patterns are known, and 70% of pathogenic *DES* carriers have some form of cardiac involvement.

Desmin is most commonly associated with myofibrillar myopathy I and DCM with a propensity for conduction system disease and arrhythmia (aDCM). *DES* was first considered a possible ACM candidate gene in 2009; a missense variant found in 27 patients expressed a fully penetrant, variable cardiac phenotype (high-grade AV block, arrhythmias originating from the RV and RV failure) [79]. Further family studies identified more *DES* variants in ACM patients including variants associated with predominant LV involvement (ALVC form) [80], SCD [81], and mixed cardio-skeletal myopathies [82]. A number of in vitro functional alteration studies involving mutant cells suggest a moderate association between *DES* and ACM; main findings include evidence of fibrosis and disruption of the ID and filaments [80,81,83].

The term ‘desminopathy’ is sometimes used for phenotypes caused by pathogenic variants in *DES* which result in skeletal muscle disorders with inclusion bodies formation, weakening of the desmin intermediated filament cytoskeleton, disruption to subcellular organisation of organelles and myofibrillar degradation. There is marked clinical heterogeneity as well as intra- and inter-familial variation, with the same mutation. Phenotypes can include isolated cardiomyopathy to a range of skeletal muscle disorders (limb girdle, scapuloperoneal, distal myopathies) and varying respiratory involvement [84]. CMR studies, even in asymptomatic individuals, often show early presence of late gadolinium enhancement (LGE) [85].

#### 3.2.4. Filamin C (FLNC) (Encoded by *FLNC*)

Filamin C is an actin cross-linking protein found only in striated muscle, important for structural cell stability [64]. Variants in *FLNC* were initially shown to be associated with skeletal myofibrillar myopathy [86], restrictive cardiomyopathy [87] and possibly hypertrophic cardiomyopathy (missense variants causing protein aggregate toxicity). More recently this gene, particularly truncating variants, have been associated with a high SCD risk ALVC [88,89], ventricular arrhythmias and a characteristic LGE pattern on CMR (Table 2) [39,43]. Furthermore, a distinct immunohistochemical phenotype has been reported in these variants with altered protein localisation in the ID [43]. Further functional studies are needed to elucidate the mechanistic links resulting in arrhythmia and structural changes.

The high incidence of SCD in these families led to recommendations to consider prophylactic implantable cardioverter defibrillators (ICD) for SCD prevention: the 2019 HRS ACM document provides a class IIa/level of evidence C recommendation for a primary prevention ICD in *FLNC* pathogenic variant carriers with LVEF < 45% [1]. Reports from large cohorts are still needed to support this.

#### 3.2.5. Tight Junction Protein 1 (TJP1) (Also Known as Zona Occludens 1) (Encoded by *TJP1*)

*TJP1* encodes the ZO-1 (zonula occludens-1) protein, a multi-functional scaffolding protein that localises to the ID of cardiomyocytes. ZO-1 has been documented to interact with a series of related proteins, including connexin43, N-cadherin, αT-catenin, and actin, and thus represents an intriguing candidate for ACM. Limited evidence supports the pathogenic role of *TJP1* pathogenic variants in ACM. Borteli et al. used whole exome sequencing (WES) to identify a variant in one ARVC family, with further rare variants identified in additional unrelated ARVC cohorts [44]. In silico tools predicted these variants to be deleterious and affect highly conserved amino acids, and either cause local unfolding and promote structural rearrangements of the GUK (guanylate kinase) domain, or impair the function of the disordered region. Case-control studies provided evidence for enrichment with *TJP1* variants in ACM patients compared with controls, supporting the causality of *TJP1* variants in ACM [44]. Further evidence from larger cohorts for the role of *TJP1* gene as a disease causing in ACM is still needed.

#### 3.2.6. N-Cadherin (CDH2) (Also Known as Cadherin-2) (Encoded by *CDH2*)

N-cadherin, encoded by *CDH2* (part of the Cadherin gene family) is a large transmembrane adherens junction (AJ) protein (906 residues, 99.8 kDa) that connects actin filaments in neighbouring cardiomyocyte sarcomeres [52]. Functions include providing strength and stability to cardiac tissue, calcium-ion-dependent adhesion, as well as playing a role in left-right asymmetry, stability of gap junctions, development of the nervous system and the formation of cartilage and bone. The *CDH2* gene is large with 16 exons and >200 kb and undergoes alternate splicing resulting in multiple transcript variants, with one of these involved in a proteolytically processed preproprotein, which generates a calcium-dependent cell adhesion molecule and glycoprotein. In 2017, WES revealed a novel *CDH2* variant in an ARVC three-generation South African family [90]; subsequent screening of a separate ARVC genotype-negative cohort identified another *CDH2* variant [91]. *CDH2* is a strong candidate gene for ACM; AJs and desmosomes are closely interacting structures and furthermore, PKG, a central desmosomal protein, is known to associate with cadherins [92].

#### 3.2.7. Catenin Alpha 3 (CTNNA3) (Encoded by *CTNNA3*)

Alpha T-catenin 3 is an ID adhesion molecule known to interact with implicated ACM molecules, plakophilin-2 and both N- and E-cadherins [52,93]. Despite this, limited evidence is currently available implicating *CTNNA3* in ACM. In 2013, Van Hengel et al. analysed the *CTNNA3* gene in 76 desmosomal-gene negative ARVC patients; two variants were identified in two probands [45]. The same group used cell cultures and yeast two-hybrid assays to identify abnormal α-T-catenin dimerization as a possible pathophysiological mechanism [45]. One study found *CTNNA3* knockout mice exhibit a progressive cardiomyopathy with increased arrhythmic propensity following ischaemia [94].

#### 3.2.8. Transmembrane 43 (Also Known as LUMA) (TMEM43) (Encoded by *TMEM43*)

TMEM43 is a nuclear membrane organiser protein known to bind with lamin and other nuclear proteins. Merner et al. reported using positional mapping to isolate a 2.36 Mb disease region in 15 unrelated ARVC families from a genetically isolated founder population in Newfoundland, Canada [95]. Bi-directional sequencing identified one rare variant in *TMEM43* (p.S358L) [95]. Validation from segregation studies, animal models, expression studies and in vitro assays support a strong variant-disease relationship. Functional studies have revealed that the p.S358L variant alters intercalated disc protein expression and reduces conduction velocity in ACM [96], but the pathobiology of *TMEM43* disease is still poorly understood and needs to be further investigated. Independent pathogenic variants associated with ACM have also been reported in other populations [97,98]. Dominguez et al. recently described the p.S358L mutation within three unrelated non-Newfoundland Spanish families [46]. Affected patients exhibited an ARVC-5 phenotype similarly described in the Newfoundland population; a fully penetrant, biventricular with LV predominance ACM with characteristic ECG findings (lower voltages in V3 and prolonged QRS duration in right precordial leads) and a high predilection for SCD in males [46,95]. In addition, female carriers known to participate in vigorous exercise were found to have a more adversely expressed phenotype [46].

#### 3.2.9. Phospholamban (PLN) (Encoded by *PLN*)

Phospholamban is a 30 kDa homopentamer encoded by the *PLN* gene, which is small (1 intron, 1 exon and 13.2 kb) and well conserved across species [99]. This protein is involved in calcium regulation and is a major substrate for cAMP-dependent protein kinase in cardiac muscle. In the unphosphorylated form it inhibits sarcoplasmic reticulum Ca^2+^-ATPase (SERCA) and in the phosphorylated form it no longer inhibits SERCA, which then pumps cytosolic Ca^2+^ back to the sarcoplasmic reticulum, and thereby relaxes muscle [100]. Pathogenic variants in *PLN* are classically associated with aDCM. In 2012, Van der Zwaag et al. screened a cohort of 97 ARVC and 257 DCM unrelated Dutch index patients for *PLN* mutations. Interestingly, 12% of ARVC patients and 15% of DCM patients had a specific *PLN* mutation (*PLN* p.R14del), which was later found to originate from a single founder over 575 years ago [101]. This variant is carried by 1 in 1500 Dutch people, and has also been reported in the US, Canada, and other European countries. Delayed afterdepolarisations (DAD) are a recognised arrhythmic manifestation of intracellular calcium overload, can occur after full repolarisation, and are induced by spontaneous calcium release. Thus, an important mechanistic link of initiation of arrhythmia in ACM is due to loss-of-function mutations in SERCA, remaining unphosphorylated and thus inhibiting SERCA, leading to build up of intracellular Ca^2+^ and DAD [102].

In addition to the typical TWI seen in precordial leads in ARVC, patients with *PLN* p.R14del have low voltage QRS [101]. However, the degree of ventricular involvement is highly variable with both ARVC, BivACM and aDCM. Patients tend to manifest arrhythmias in the 2nd and 3rd decades of life. There are discrepancies between human clinical, iPSC and murine studies with current efforts focused on further elucidating the mechanisms of arrhythmia and structural abnormalities, as well as developing novel therapeutics [103].

The high incidence of SCD in these families led to recommendations to consider prophylactic implantable cardioverter defibrillators (ICD) for SCD prevention: the 2019 HRS ACM document provides a class IIa/level of evidence B recommendation for a primary prevention ICD in *PLN* pathogenic variant carriers with LVEF < 45% or NSVT [1]. Reports from large cohorts are still needed to support this.

#### 3.2.10. Ryanodine Receptor 2 (RYR2) (Encoded by *RYR2*)

RYR2 is a sarcoplasmic reticulum calcium release channel previously reported in association with ARVC [104]. However recently, this gene-disease association is increasingly questioned due to contradictory evidence [105], the presence of ARVC-associated variants in reference alleles, incomplete inheritance information and strong co-association of catecholaminergic polymorphic ventricular tachycardia (CPVT) (a cardiac ion channelopathy characterised by adrenergically-mediated arrhythmias in individuals with structurally normal heart) [33,104,106]. Interestingly, deletion of the exon 3 in *RYR2* has been associated with an extended phenotype of DCM, sinoatrial node dysfunction, atrial fibrillation, and atrial standstill combined with CPVT [107].

#### 3.2.11. Voltage-Gated Sodium Channel (Na_v_1.5) (Encoded by *SCN5A*)

Pathogenic variants in *SCN5A* were shown to result in a disruption of the voltage-sensing mechanism of the voltage-gated sodium channel subunit Na_V_1.5, which conducts the I_Na_ current [108]. Defective Na_V_1.5 results in electrical alterations and myocardial dysfunction. Loss-of-function variants in this gene cause Brugada syndrome (BrS) and/or progressive cardiac conduction disease, whereas gain-of-function variants typically produce long QT syndrome (LQTS) type 3 phenotype, or rarely familial atrial fibrillation [33]. Infrequently, both gain-of-function and loss-of-function variants in *SCN5A* can lead to an aDCM phenotype, usually with no apparent macroscopic fibrosis on cardiac imaging; patients often exhibit conduction disturbances at different levels as well as a high propensity to ventricular arrhythmias [108,109]. The heterogeneity of *SCN5A* mutations associated with DCM, and the heterogeneity of phenotypes renders the mechanistic explanation of *SCN5A*-mediated DCM challenging. As such, whether the DCM phenotype is a primary manifestation of the *SCN5A* genetic defect or for example a consequence of frequent ventricular arrhythmias, is yet to be answered [72,109].

In 2017, te Riele et al. used WES to discover a missense variant in *SCN5A* in one of six unrelated desmosomal gene variant-negative ARVC patients. Cohort validation in 281 ARVC patients found a *SCN5A* putative pathogenic variant frequency of almost 2%. Cellular functional characterisation of one variant found reduced sodium current and reduced Na_v_1.5 and CDH2 interaction in the ID. Although Na_v_1.5 is known to interact with the mechanical junction proteins, *PKP2* and *CDH2* [49], and *PKP2* mutations can be associated with a sodium channelopathy-type phenotype [49], mechanisms linking *SCN5A* variants to clear ARVC phenotype remain poorly investigated [109].

#### 3.2.12. Transforming Growth Factor Beta 3 (TGFβ3) (Encoded by *TGFB3*)

TGFβ3 is a cytokine protein associated with tissue development, homeostasis and fibrosis induction [110]. In 1994, parametric linkage analysis in five ARVC families identified *TGFB3* as one of six possible candidate genes in a disease region [111]. However, sequencing of coding regions of these six genes did not identify any pathogenic variants. Over 10 years later, Beffagana et al. identified two variants in untranslated regions (UTR) of *TGFB3*; a 5′ UTR mutation segregating in a large ARVC family and a 3′ UTR mutation associated with an unrelated ARVC patient [110]. Transfection studies in a murine myoblast cell line found that both variants caused greater luciferase reporter activity compared to controls [110], suggesting greater TGFβ3 expression. It has been suggested that genetic defects in *TGFB3* induce myocardial fibrotic response through promoting the expression of extracellular matrix genes and inhibiting extracellular matrix degradation by suppressing the activity of matrix metalloproteinases [110,112]. Of note, TGFβ3 is the only secreted protein implicated in the pathogenesis of ACM so far; should its causality in ACM be extensively validated in future studies, it will suggest a mechanism involving paracrine and autocrine signalling.

#### 3.2.13. RNA-Binding Motif Protein 20 (RBM20) (Encoded by *RBM20*)

RNA-binding motif protein 20 is a splicing factor involved in regulating constitutive and alternative splicing of a number of key cardiac genes (sarcomeric, calcium regulation and ion regulation genes) [113,114]. Pathogenic *RBM20* variants resulting in loss-of-function lead to missplicing of sarcomeric and calcium-handling genes, implicated in DCM (*TTN* missplicing considered to be the major contributing mechanism), and are associated with an aggressive early-onset phenotype with an increased predilection to cardiac transplantation, severe arrhythmia and SCD [114,115,116]. Brauch et al. identified distinct heterozygous missense mutations in exon 9 of *RBM20* in 2 large families with DCM [117]. Moreover, van den Hoogenhof et al. compared the arrhythmia burden of DCM patients with *TTN* mutations (*n* = 22) and DCM patients with *RBM20* mutations (*n* = 18). Most notably, they found 44% of *RBM* carriers had sustained ventricular arrhythmia compared to 5% in the *TTN* group. From these results, they hypothesised that other mechanisms apart from *TTN* splicing may explain the RBM20 phenotype. Using *RBM20* knockout murine and cardiomyocyte models, they discovered intracellular calcium overload as a mechanism due to missplicing of CamkIIδ (another RBM20 target gene) [113].

Parikh et al. interrogated the 14 exons of *RBM20* by comparing 74 patients with cardiomyopathy phenotype to variants in the general population, and identified exon 11 as a new pathogenic hot spot. The phenotype included a high prevalence of atrial fibrillation (AF), non-sustained VT, ICD discharge, and SCD events. Thus, *RMB20* is a complex gene involved in regulation of multiple sarcomeric and calcium-regulatory genes, with a high propensity for arrhythmias and structural abnormalities.

#### 3.2.14. BCL2-Associated Athanogene 3 (BAG3) (Encoded by *BAG3*)

BAG3 is a co-chaperone of the Heat shock 70 kDa protein 8, which functions to facilitate folding of newly translated proteins and misfolded proteins, and degrade protein aggregates [118]. In the last decade, *BAG3* variants have been implicated in DCM. One study used WES and genome wide analysis of copy number variation to identify a large deletion in *BAG3* in a 3-generation DCM family [119]. Further variants were identified in 7 unrelated probands from a 311 DCM genotype-negative cohort [119]. Observations from experimental data are inconclusive and limited; one *BAG3* knockdown zebrafish model observed variable phenotypes including heart failure and a skeletal myopathy [119]. Another model from DCM-related mutant cardiomyocytes identified disrupted Z-discs and stress-induced apoptosis [120]. Of note, *BAG3*-related DCM is associated with a late onset, severe progressive heart failure (HF) phenotype, worse outcomes in individuals of African ancestry and a higher likelihood of terminal DCM requiring cardiac transplantation [118,121,122,123]. Despite its characteristic late-disease onset, the disease penetrance in individuals >40 years of age reaches 80% [118].

#### 3.2.15. NK2 Homeobox 5 (NKX2-5) (Encoded by *NKX2-5*)

*NKX2-5* encodes a homeodomain-containing transcription factor essential for cardiac morphogenesis. Pathogenic variants in *NKX2-5* cause diverse cardiac phenotypes with variable expressivity [124], most commonly progressive atrioventricular block and congenital heart defects (especially secundum atrial septal defect (ASD)) [125]. *NKX2-5* variants have also been implicated in adult-onset DCM [126] and ventricular arrhythmias [127]. The p.Phe145Leu variant in *NKX2-5*, carried by 1 in 7100 Icelanders, was reported to cause severe adult-onset familial DCM with very high penetrance and an increased risk of SCD [128].

## 4. Effect Modifiers

**Figure 21:**
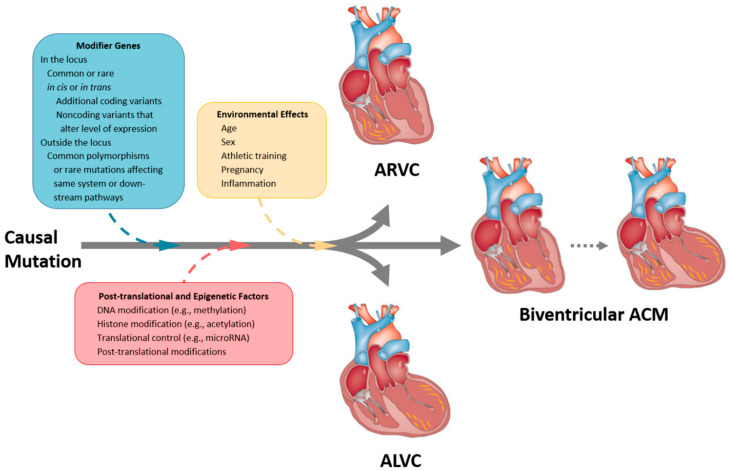
Schematic view of the possible modifier genes, post-translational, epigenetic, and environmental factors contributing to the pathogenesis of ACM including ARVC, ALVC, and biventricular ACM. Modified from Watkins et al. (2011) [131]. ACM = arrhythmogenic cardiomyopathy; ALVC = arrhythmogenic left ventricular cardiomyopathy ARVC = arrhythmogenic right ventricular cardiomyopathy.

ACM is typically an autosomal dominant disorder with age-related reduced penetrance and highly variable expressivity [129]. Around 35–50% of patients are found to have no identifiable pathogenic variant (otherwise known as gene-elusive or genotype-negative), suggesting unidentified genes and additional disease pathways might be involved. Furthermore, individuals who carry ACM-associated variants may show no disease expression, indicating that other variables and effect modifiers might be at play [2,130]. A number of disease modifiers have been recognised in ACM, and; these are summarised in Figure 21. We discuss in more detail two important effect modifiers below:

### 4.1. Inflammation

The role of inflammation in ACM is currently unclear. Myocardial inflammation is very much present in ACM; two-thirds of ARVC cases show T-cell infiltrates at autopsy; however, it is unknown if this is a primary phenomenon or reactive to ACM pathology [22]. Numerous reports have implicated proinflammatory mediators in ACM. Multiple inflammatory cytokines have been found at higher levels in serum from ARVC patients when compared to controls [132,133]. In addition, further studies have found marked local myocardial production and secretion of proinflammatory mediators in ARVC samples and models [132,134], with one study interestingly reporting a correlation between expression levels of two specific cytokines and ejection fraction in *DSG2*^mut/mut^ mice [134]. Activation of nuclear factor-κB (NFκB), an inflammatory-response transcription factor, has been characterised in both in vitro and in vivo models of ACM; of note, an inhibitor of NFκB was recently found to markedly reduce inflammatory cytokine levels whilst mitigating the development of ACM disease features [134].

Of note, an increasing number of ACM cases presenting with myocarditis-like ‘hot phases’ are being reported [135,136,137]. These episodes may mark the first development of disease or disease progression [138]. More attention is being paid to the theory that a ‘genetically vulnerable myocardium’ may be susceptible to myocarditis; however, data remain inconclusive thus far [22]. Protonotarios et al. retrospectively analysed 16 ARVC patients referred for 18F-fluorodeoxyglucose positron emission tomography (FDG-PET)—a validated technique for detecting myocardial inflammation in suspected myocarditis. Despite a number of study limitations, the group demonstrated 36% of their ARVC patients on FDG-PET had active myocardial inflammation [139]. Lopez-Ayala et al. performed a retrospective analysis of the medical records of 131 ARVC patients and 64 phenotype-negative genotype-positive relatives [140]. They found seven patients who had previously presented with acute myocarditis (one being from the phenotype-negative group); five of these patients carried *DSP* variants and all of these patients were relatives from a single ancestor [140]. A recent study reported on monozygotic twins who presented with myocarditis at ages 17 and 18; further investigation found extensive LGE pattern only involving the LV and a *DSP* variant in both twins [141]. This supports the hypothesis of a genetically susceptible myocardium, with myocarditis being an initial trigger or ‘hot’ phase for the disease.

Interestingly, in addition to clinical overlap, pathological overlap exists between ACM and cardiac sarcoidosis, a granulomatous inflammatory condition of the myocardium. Aberrant distribution of desmosomal proteins at the ID has been reported in sarcoidosis, whilst cytokines implicated in sarcoidosis have been found to cause loss of junctional plakoglobin in cardiac myocytes [132]. Increasing evidence of autoimmunity has also been recently reported with two studies identifying anti-heart, anti-intercalated disc and anti-DSG2 autoantibodies in patients with ACM [142,143]. Further study is required to understand and clinically identify specific and distinguishable inflammatory pathways seen in ACM.

### 4.2. Exercise

A strong link has been established between endurance exercise participation and an adversely expressed cardiac phenotype [144,145]. Of those who are desmosomal pathogenic variant carriers, an increased age-related penetrance of ACM, risk of ventricular tachyarrhythmias and HF is observed [144]. There is evidence to suggest exercise plays a disproportionate role in gene-elusive ACM patients; one study found this group participated in more high-intensity exercise when compared to desmosomal ACM patients [146]. Furthermore, gene-elusive patients with the most intense exercise history prior to presentation were found to present at a younger age [146]. More recently, in ACM patients with ICDs, exercise reduction has been shown to decrease ventricular arrhythmias, with gene-elusive patients gaining particular benefit [147]. Further investigation is required to understand the role of exercise in ACM, in particular in non-desmosomal carriers.

## 5. SCD Risk Prediction

Central to ACM management, is determining who requires life-saving ICDs. Without doubt, ICD insertion is always recommended for secondary prevention of SCD in patients with cardiac arrest or haemodynamically unstable VT; however, primary prevention remains a challenging and daunting task. We suggest comprehensive and continuous risk evaluation (at least every follow-up visit) of patients and family members (our recommended general approach is detailed here [148]). However, a more nuanced genotype-based approach should also be considered; particularly as a significant proportion of patients have no identifiable preceding symptoms pre-SCD. A greater understanding of specific pathogenic variant and family-related phenotypes is important in establishing robust ICD protocols. The Newfoundland ICD protocol specific to *TMEM43*-p.S358L carriers in their population is an example of an effective genotype-formulated strategy; 5-year survival has risen from 65 to 95% in males and from 85% to 97% in females [149].

## 6. Translational Perspectives and Future Directions

Following numerous breakthrough discoveries in the field of ACM, current understanding of this disease is changing rapidly with evolving concepts in genetics and molecular biology. The complex genetic nature of ACM and its genotype-phenotype associations has resulted in a broader definition of the disease; however, translation of genetic findings to clinical practice requires an integrated approach that aims to redefine ACM at a higher resolution.

The current research era has made available new tools for studying the genetics of ACM, among others WES and whole genome sequencing, multi-omics technologies and human induced pluripotent stem cell cardiomyocytes (hiPSC-CMs). These tools provide a platform for unravelling the complex molecular interactions underlying ACM and for in vivo drug testing. In parallel, extensive long-term, longitudinal characterisation of ACM cohorts with integration of deep phenotyping modalities enables recognition of new common and rare genotype-phenotype associations, thereby facilitating the recognition of more benign and more severe populations. It is therefore plausible that increased understanding of these gene variant-phenotype associations and development of individualised therapies will move clinical care towards an integrated genotype-mechanism-phenotype approach, fulfilling the premise of precision medicine in ACM and improving disease outcomes in ACM families. We provide a list of research priorities in Table 3.

WES and whole genome sequencing (WGS) are the research standard for novel variant and new gene discovery and are particularly important in identifying causal genes in gene elusive ACM patients (35–50%). However, a major obstacle in correctly determining a variant’s pathogenicity is the availability of reliable functional and biochemical characterisation of genetic variants (Table 4 describes different experimental techniques).

The 2015 American College of Medical Genetics and Genomics and the Association for Molecular Pathology (ACMG-AMP) proposed a standardised classification system that grades variants pathogenicity in five classes: Pathogenic (Class 5) >95%, Likely Pathogenic (Class 4) >90%, Variant of Uncertain significance (Class 3) 10–90%, Likely Benign (Class 2) <10%, and Benign (Class 1) <5% [151]. The ACMG-AMP also proposed guidance on how to evaluate evidence for a variant’s pathogenicity, important variant and gene characteristics are taken into account; these include variant function, gene function, family co-segregation with disease, allele frequency in the general population and variant type. Different types of data and evidence are evaluated, including population data, computational and predictive data, functional data from basic science studies, segregation data, de novo data, and allelic data [151]. This guidance provides a universal framework for standardisation amongst laboratories and individual healthcare institutions; however, one emerging theme contributing to incorrect classification is lack of specialty expert input. In response to this, the ClinGen Cardiovascular Clinical Domain Working Group have formed an inherited cardiomyopathy expert panel, made up of international experts in clinical cardiology, clinical research, molecular diagnostics, genetic counselling and genomic medicine, and begun to adapt the ACMG-AMP framework for use in inherited cardiomyopathies such as ACM. They recently published recommendations for variant classification for *MYH7*-associated inherited cardiomyopathies [152]. This multi-specialty integrated approach to variant classification is needed on local, national and international levels, to maximise patient benefit. 

In the oncological world, creating humanised animals—model avatars—of the specific genetic defect of the cancer, and testing anti-neoplastic agents is being used to deliver precision medicine [153]. Recently, an in vivo rat-hiPSCs-CM model derived from ACM patients and able to reproduce their genotypic status, has been found to model phenotypic characteristics seen in ACM [154]. This new model provides a magnified opportunity to investigate the disease mechanisms underlying specific ACM genotypes and undertake pharmacological testing within the macrocosm of a mammalian heart [155]. Modelling ACM in experimental settings provides important pathophysiological understanding, but also a possible avenue for discovery of pharmacological therapies. Recently, the compound SB216763 (a GSK3β inhibitor that prevents degradation of β-Catenin) was found to improve the cardiac phenotype of zebrafish embryos and murine models expressing different genetic mutations [156,157]. Another example, BAY11-7082 (an NFκB inhibitor) was found to prevent development of all features of disease in ACM models [134]. These promising discoveries pave the way for precision medicine therapies in the future.

**Figure 22:**
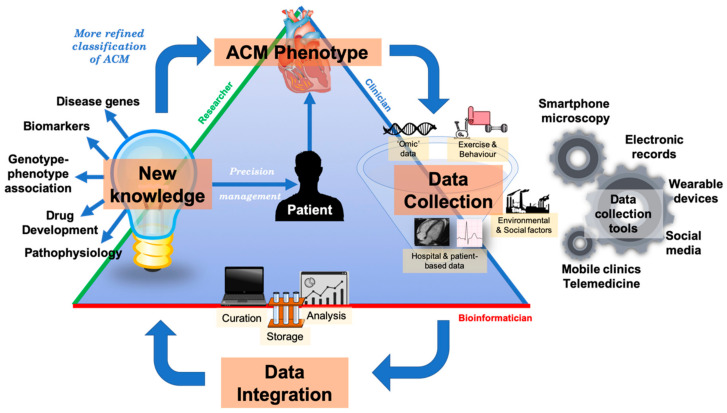
An integrated precision medicine systems model in ACM. The goal of precision medicine is to redefine the classification of diseases such as ACM into more refined classes. Multidimensional longitudinal data is collected by clinicians, clinical researchers and patients; technological advances in the last decade have provided opportunities to deeply phenotype and rapidly genotype patients—we describe some innovative data collection tools in our diagram. Data is curated, analysed and stored known as data processing by bioinformaticians using computational methods. Processed data can be used to discover new knowledge about a disease phenotype such as biomarkers, new disease genes etc. (the field of research). These insights can be translated to provide more personalised management to patients. As disease phenotypes such as ACM become more refined, e.g., from morphofunctional classes to molecular classes, more targeted therapies will be discovered. ACM = arrhythmogenic cardiomyopathy.

Bringing together research and clinical practice is vital to improve outcomes in rare and heterogenous condition such as ACM. To do this, a ‘precision medicine network’ has been proposed which connects patients, clinicians, laboratories, researchers, bioinformaticians and other stakeholders [158]. At a simplistic level, a precision medicine network is the infrastructure that facilitates communication between these parties. This enables efficient flow of data, information and new knowledge to encourage a ‘learning health system’, where clinical practice and research can synergise with the goal of improving patient outcomes—this is particularly important in rare diseases where patient cohorts are smaller and thus may require greater collaboration. For example, in determining pathogenicity of a specific variant in an ACM patient, the interface between cardiologists, geneticists, bioinformaticians, and researchers is essential to deliver the best possible care. Specialist cardiologists ‘phenotype’ patients using advanced studies such as CMR with tissue characterisation, signal-average ECGs, voltage maps, etc.; geneticists and bioinformaticians ‘genotype’ patients and their families and translate that data into clinical information and researchers provide validatory information by performing basic science and clinical research to generate new knowledge (see Figure 22). The initial reluctance of the adoption of telemedicine due to bonafide concerns over privacy and confidentiality has been surpassed with recent world events and rapid implementation across the globe. This has been on the whole well received by both patients and clinicians [159]. One of the major limitations of evaluating novel variants is cascade clinical and genetic screening in family members who live far from the evaluating centre; telemedicine now allows families to be evaluated in a single centre and focus on reclassifying variants which are often ‘private’ to families. This, with shared electronic health records, mailable kits for collecting biospecimens will facilitate participation in research to discover novel biomarkers and genetic variants. Social media-based recruitment has also facilitated recruitment of probands and family members who may be in an area without specialist genetic cardiology services, such as in our Mayo-Cambridge Registry (https://clinicaltrials.gov/ct2/show/NCT03049254). Recruitment can now also be done via video-link or an online portal with digital consent as used for the National Institutes of Health (NIH) All of Us project [160].

## 7. Conclusions

With technological advances in genomic sequencing, diagnostic modalities, and research tools, opportunities to understand ACM at a deeper level are being realised. The classification of ACM and nosology of cardiomyopathies are likely to become more refined as we piece together the complex genotype-phenotype relationship. Thus, an integrated multi-speciality approach involving clinicians, bioinformaticians, researchers and most importantly, patients is required to translate these molecular insights into clinical practice. Precision clinical care requires an in-depth and nuanced understanding of the genetics of ACM, whilst also understanding the correlating deep phenotypic characteristics, to ensure optimal timely and targeted intervention.

## Figures and Tables

**Figure 4:**
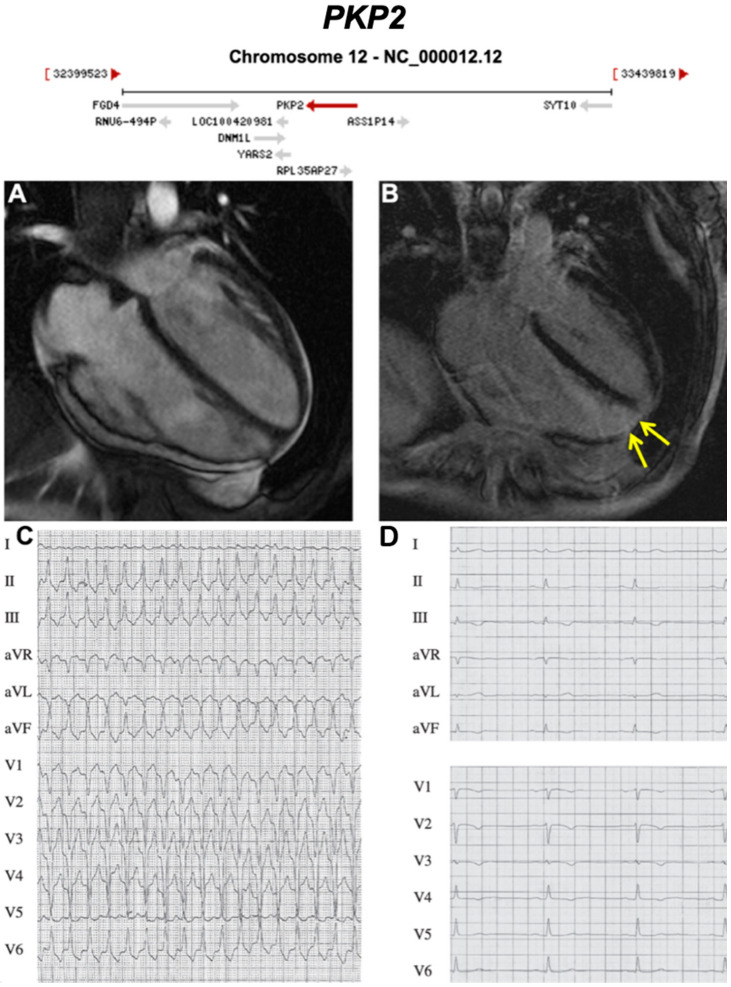
Exemplar MRI and ECG finding found in ACM patients with *PKP2* mutations. (**A**) CMR of a patient with *PKP2* c1664*del* T mutation reveals moderate enlargement of the right ventricle; (**B**) yellow arrow shows apical fibrosis in LGE mode;.(**C**) 12-Lead ECG of same patient showing ventricular tachycardia with left bundle branch block morphology and inferior axis; (**D**) resting 12-Lead ECG upon admission to our hospital showing sinus rhythm, and T-wave inversion is present in V1-V4. Reproduced with permission from Trenkwalder et al. (2015) [34].

**Figure 5:**
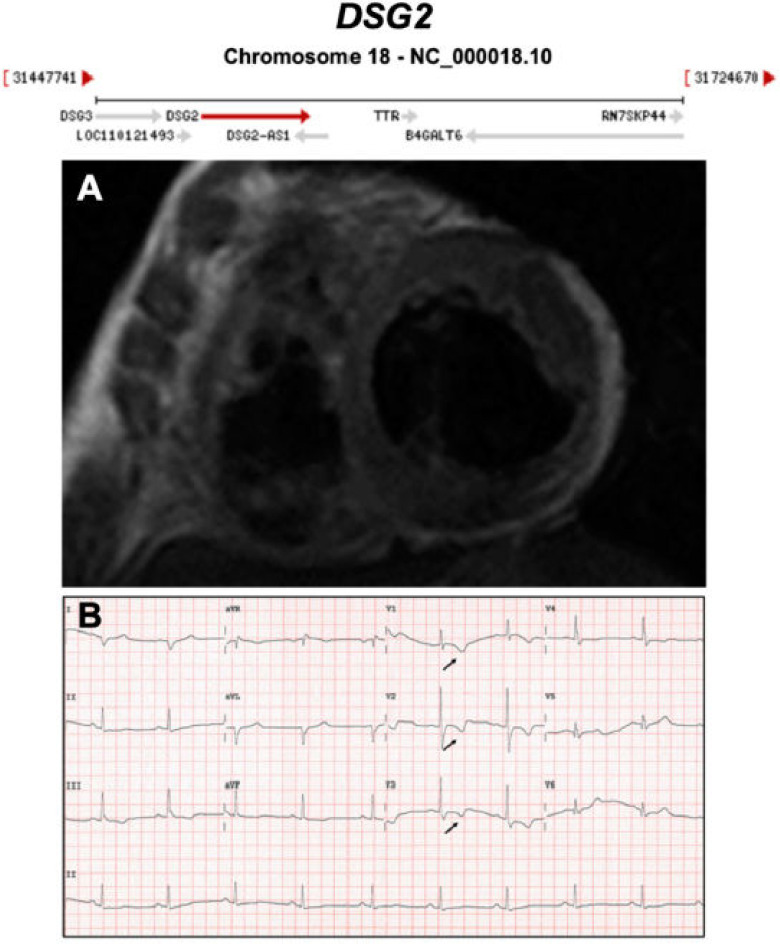
Exemplar MRI and ECG finding found in ACM patients with *DSG2* mutations. (**A**) CMR of a patient with *DSG2* p.Leu237Ter mutation showing dilation of both ventricles; (**B**) a representative ECG of the same patient showing T wave inversion in V1-V3. Reproduced with permission from Chen et al. (2020) [35].

**Figure 6:**
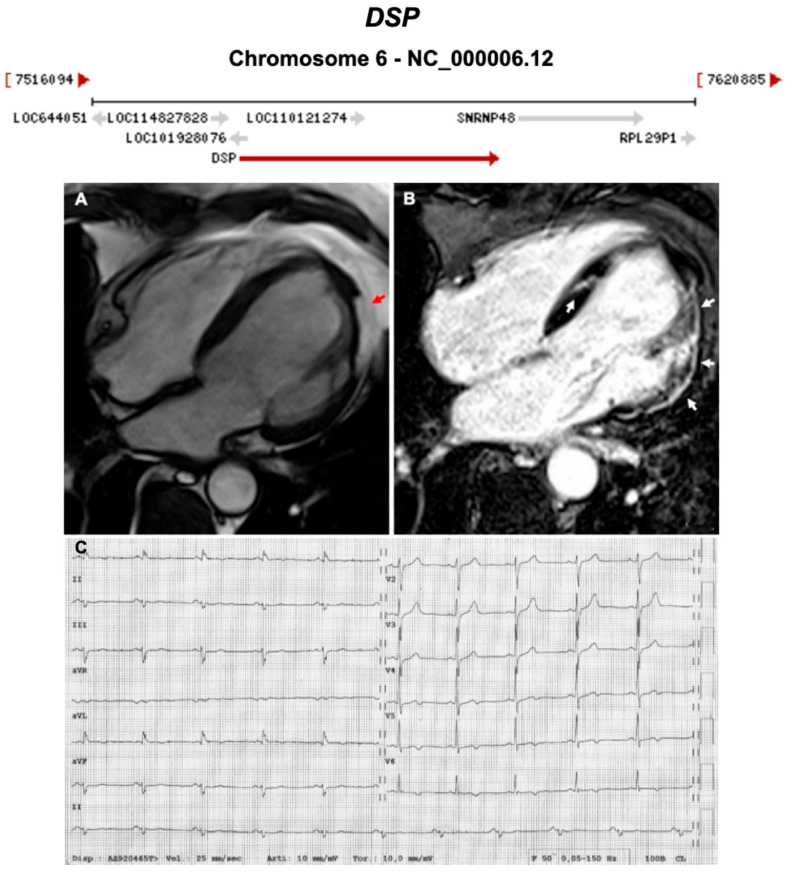
Exemplar MRI and ECG finding found in ACM patients with *DSP* mutations. (**A**) CMR of a patient with *DSP* mutation showing left ventricular lateral wall fatty infiltration (red arrow); (**B**) LGE MRI of the same patient showing fibrosis/scaring of both left ventricular lateral wall and septum; (**C**) 12-lead ECG showing T-wave inversion in V5-V6 (anterolateral leads). Reproduced with permission from Mattesi et al. (2020) [36].

**Figure 7:**
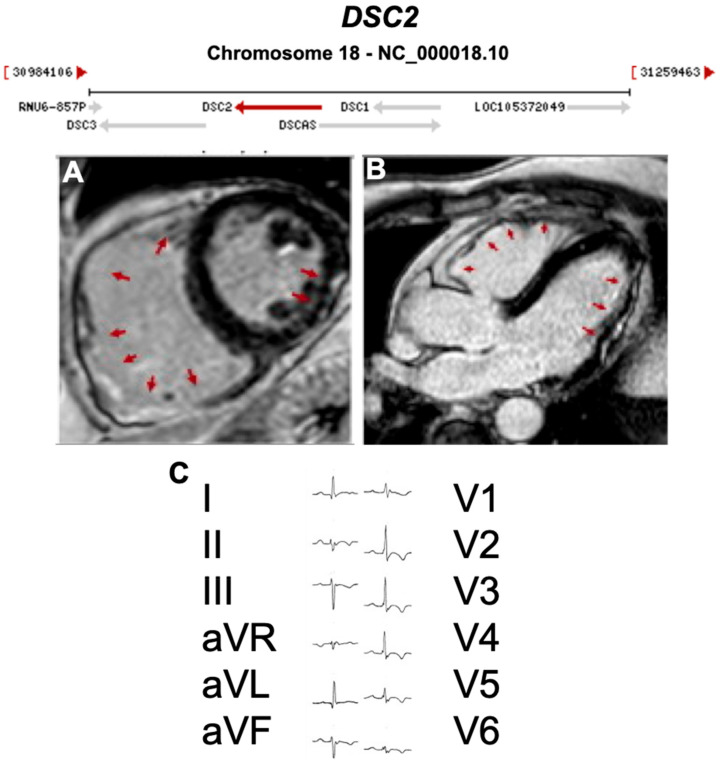
Exemplar MRI and ECG finding found in ACM patients with *DSC2* mutations. (**A**,**B**) CMR of a patient with *DSC2* p.Arg49His mutation showing biventricular involvement in the LGE mode (red arrows); (**C**) 12-lead ECG showing a reduction of R waves amplitude in left precordial leads (left involvement) and inverted T waves in V1-V6 (RV strain pattern); together they indicate biventricular involvement. Reproduced with permission from Gaido et al. (2017) [37].

**Figure 8:**
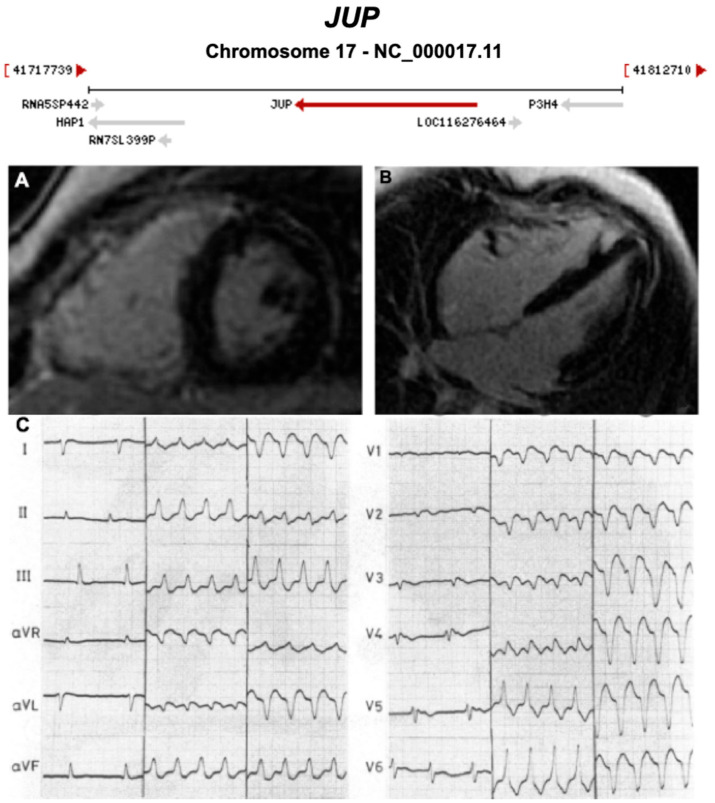
Exemplar MRI and ECG finding found in ACM patients with *JUP* mutations. (**A**,**B**) CMR of a patient with *JUP* mutation showing LGE of the RV free wall, secondary to fibrosis (white arrow); (**C**) ECG findings in a different patient with *JUP* mutation at 3 different times (separated by two vertical lines into 1. right panel = sinus rhythm with T waves inversion in V1–V6, and 2. middle and 3.left panels = ventricular tachycardia). (**A**,**B**) were reproduced with permission from Mavrogeni et al. (2012) [38]; (**C**) was reproduced with permission from Protonotarios et al. (1986) [27].

**Figure 9:**
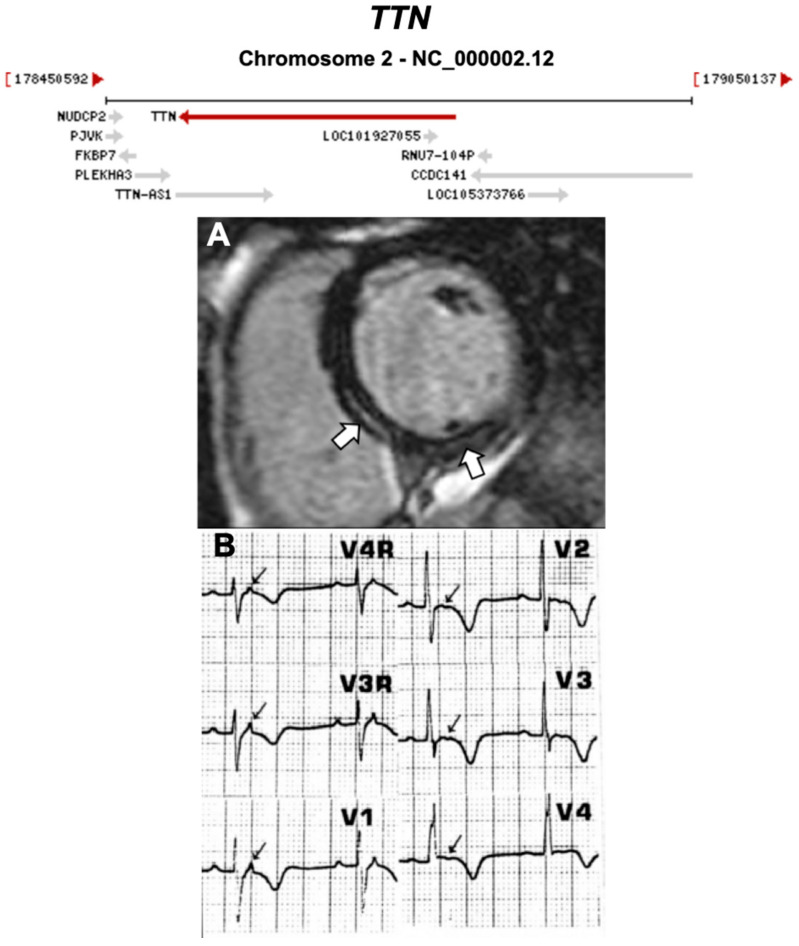
Exemplar MRI and ECG finding found in ACM patients with *TTN* mutations. (**A**) CMR of a patient with *TTN* mutation, the white arrows show mid-wall scars in the inferior septum and inferior wall; (**B**) ECG of a different patient with *TTN* Thr2896Ile mutation showing epsilon waves. (**A**) was reproduced from Augusto et al. (2019) [39]; (**B**) from Taylor et al. (2011) [40] with permissions.

**Figure 10:**
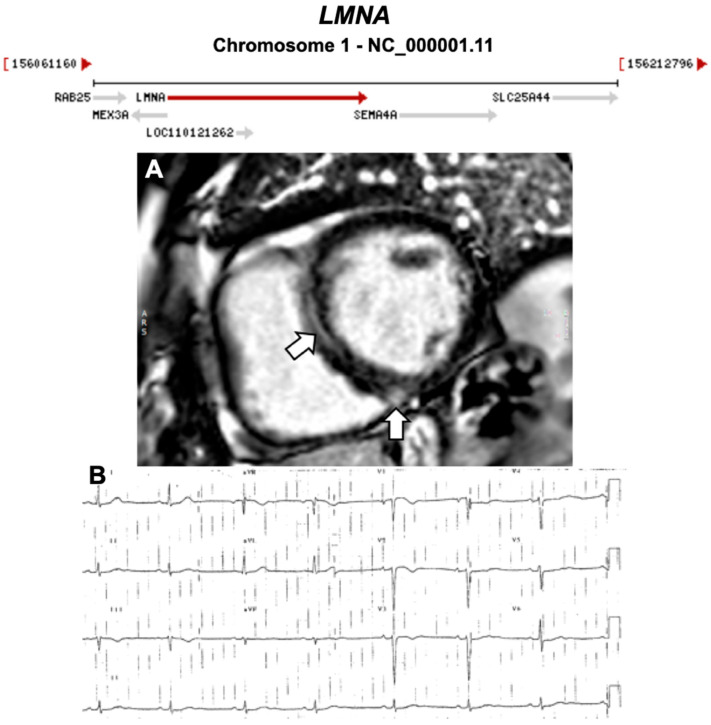
Exemplar MRI and ECG finding found in ACM patients with *LMNA* mutations. (**A**) CMR of a patient with *LMNA* mutation showing a mid-wall (LGE) scar in the septum and inferior RV; (**B**) 12-lead ECG of a different patient with a p.Gly382Val *LMNA* mutation, which shows sinus bradycardia, poor R-wave progression, T-wave inversion in V1–V3 leads. (**A**) reproduced with permission from Augusto et al. (2019) [39]; and (**B**) reproduced with permission from Quarta et al. (2012) [41].

**Figure 11:**
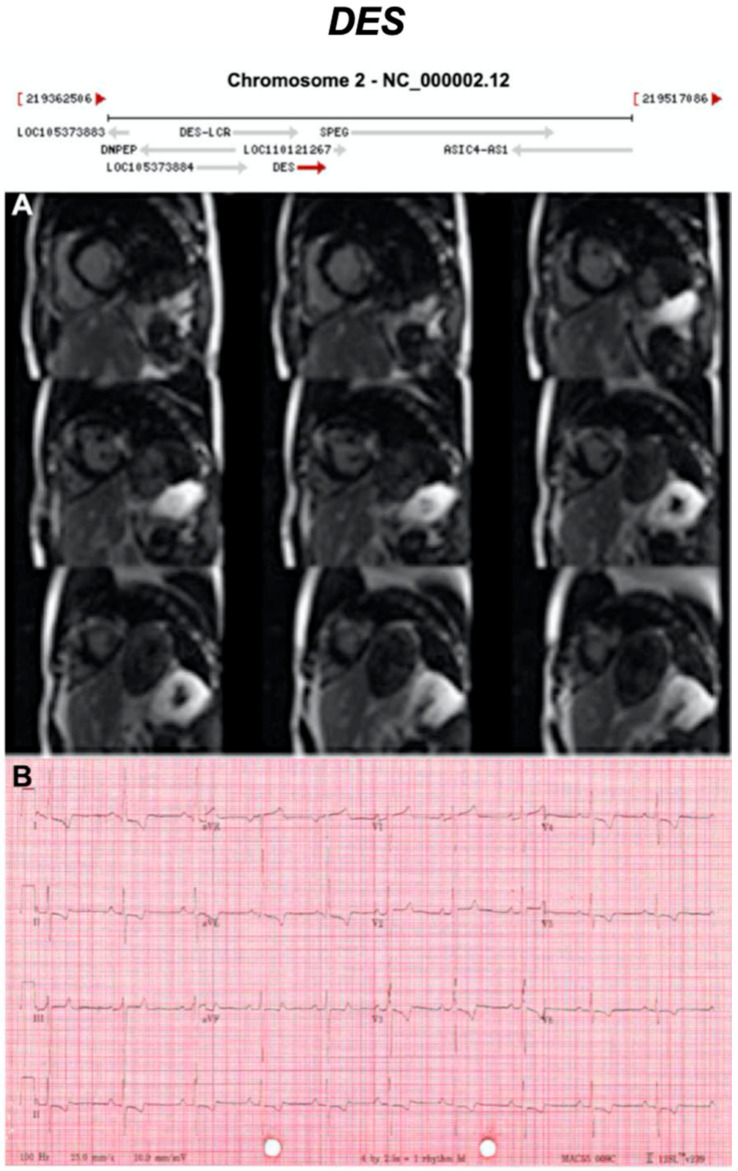
Exemplar MRI and ECG finding found in ACM patients with *DES* mutations. (**A**) CMR of a patient with *DES* mutation (deletion of guanine at position 735) showing extensive mid-wall fibrosis (LGE short-axis view); (**B**) 12-lead ECG of a different patient with p.Gly382Val mutation of the *LMNA* gene showing sinus tachycardia, inverted T waves in V1–V3 ad poor R wave progression. Reproduced from Koitka et al. (2017) with permission [42].

**Figure 12:**
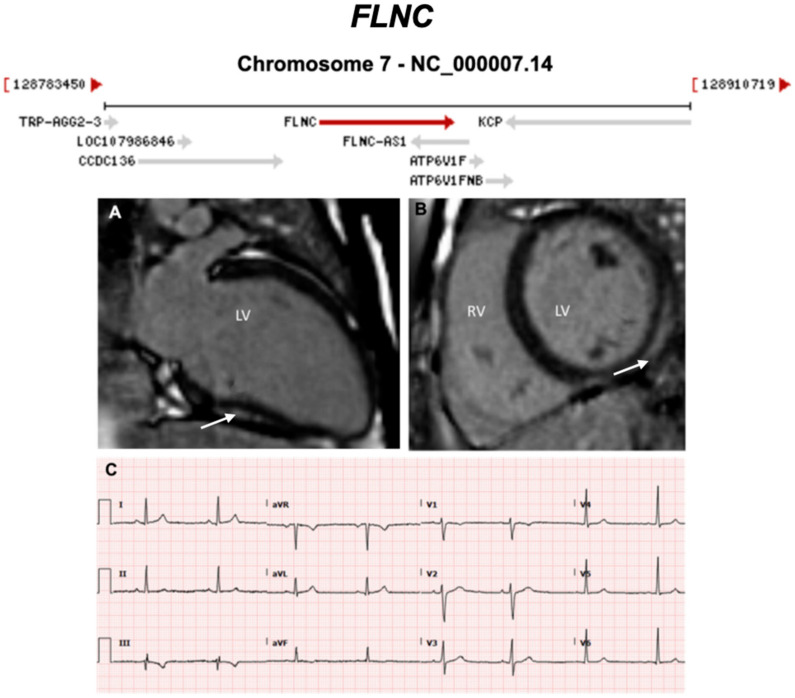
Exemplar MRI and ECG finding found in ACM patients with *FLNC* mutations. (**A**,**B**) CMR of a patient with p.Arg991X (nonsense) mutation of the *FLNC* gene; the arrows show basal lateral subepicardial fibrosis in the LGE mode (white arrows); (**C**) 12-lead ECG of the same patient showing T-wave inversion in III and aVF (inferior leads). Reproduced with permission from Hall et al. (2020) [43].

**Figure 13:**
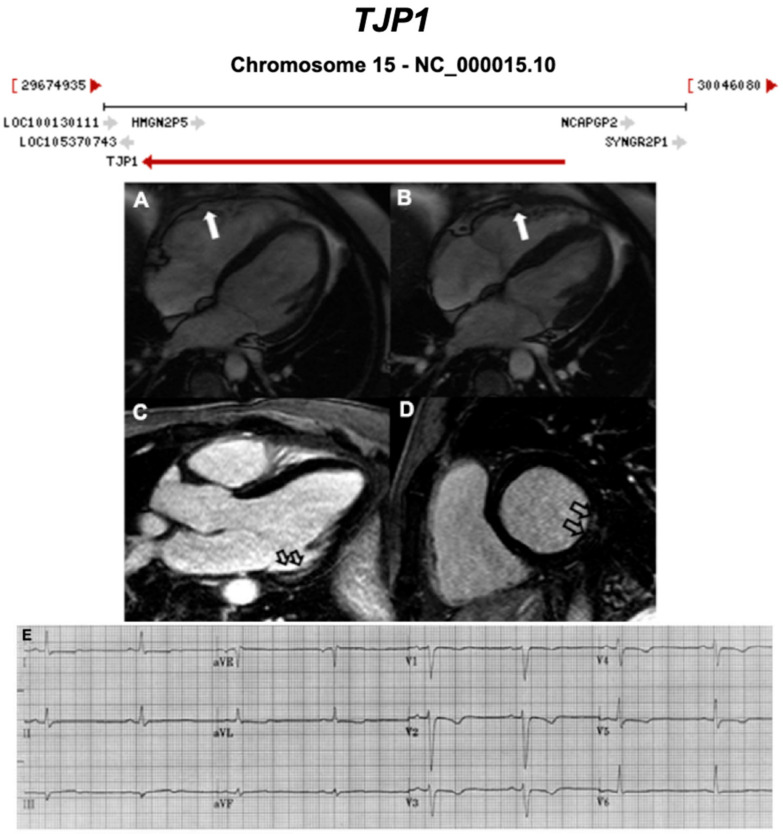
Exemplar MRI and ECG finding found in ACM patients with *TJP1* mutations. (**A**,**B**) CMR of a patient with *TJP1* p.Tyr669Cys mutation showing right ventricular dilatation (white arrows); (**C**,**D**) LGE shows mid-mural fibrosis in the inferior-lateral wall of the left ventricle (empty arrows); (**E**) 12-lead ECG of the same patient showing shows sinus rhythm, intra-ventricular conduction delay, and T wave inversion in V1 to V5. Reproduced with permission from De Bortoli et al. (2018) [44].

**Figure 14:**
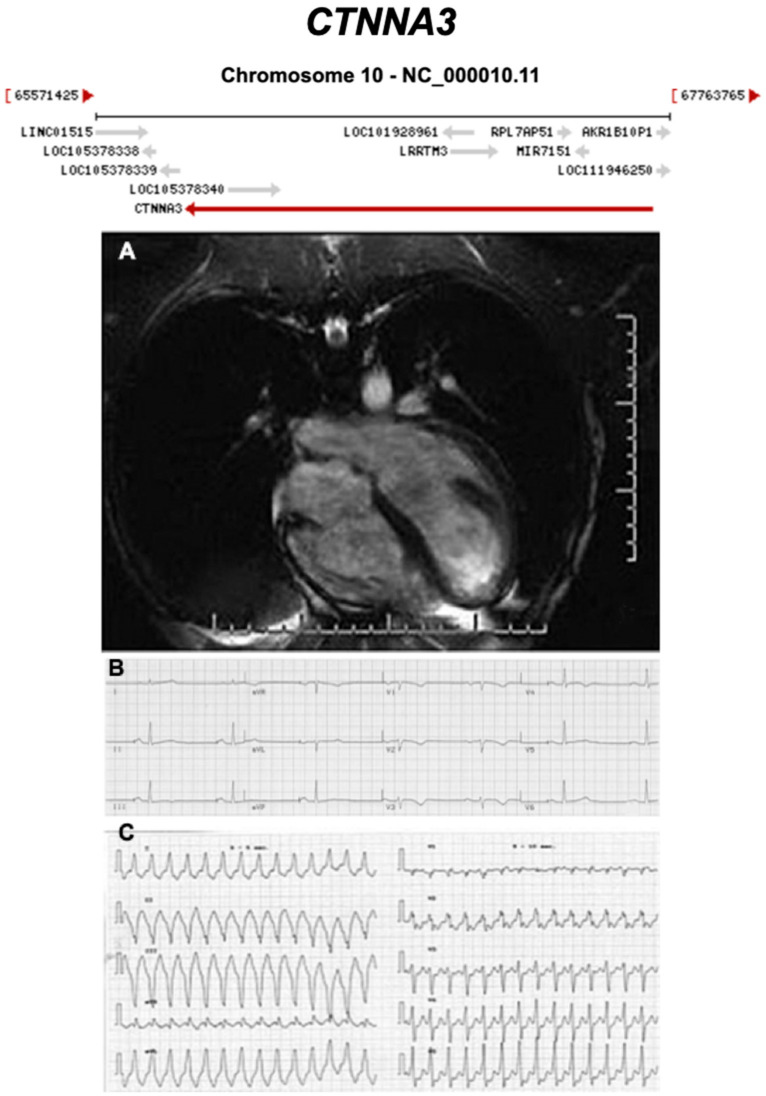
Exemplar MRI and ECG finding found in ACM patients with *CTNNA3* mutations. (**A**) CMR of a patient with *CTNNA3* p.val94asp mutation showing marked right ventricular dilatation; (**B**) 12-lead ECG of the same patient showing a first degree heart block and T-wave inversion in V1–V4; (**C**) sustained ventricular tachycardia, with left bundle-branch block and left axis deviation. Reproduced with permission from van Hengel et al. (2013) [45].

**Figure 15:**
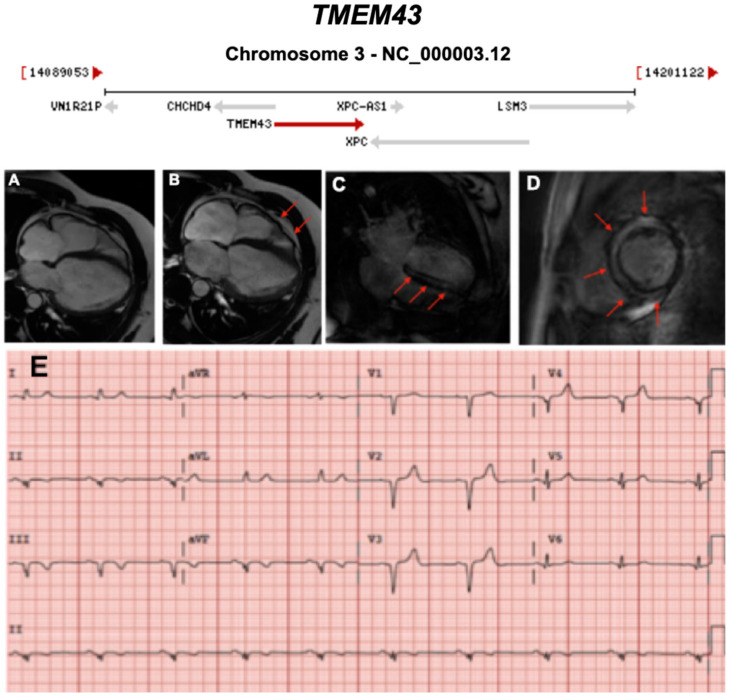
Exemplar MRI and ECG finding found in ACM patients with *TMEM43* mutations. (**A**–**D**): CMR images of a male subject with *TMEM43* p.S358L mutation. (**A**) Biventricular dilatation, wall motion abnormalities; (**B**) asynchronous contraction (red arrows); (**C**,**D**) LGE showing severe and almost concentric intra-myocardial lesions (red arrows); (**E**) a representative 12-lead ECG of a different patient with the same *TMEM43* mutation, showing poor R-wave progression with 1-mV R-wave voltage in lead V3 and widened QRS complex. Reproduced with permission from Dominguez et al. (2020) [46].

**Figure 16:**
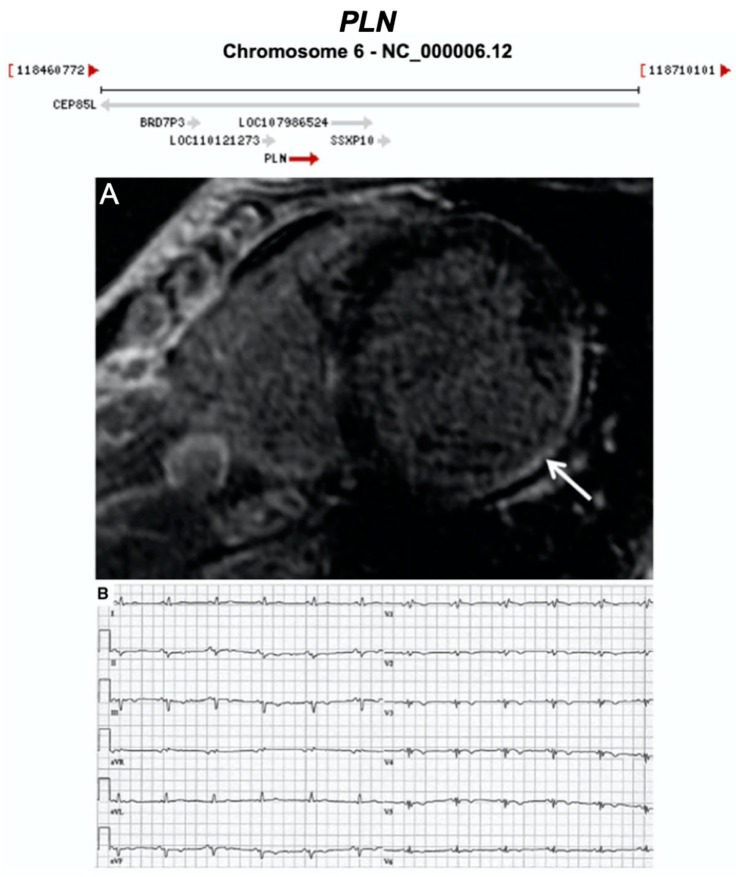
Exemplar MRI and ECG finding found in ACM patients with *PLN* mutations. (**A**) CMR image from a patient with *PLN* p.Arg14del mutation showing inferolateral wall thinning and late-gadolinium enhancement of the LV inferolateral wall; (**B**) 12-lead ECG of the same patient showing normal sinus rhythm with low voltages in all leads (<0.5 mV) and flattened or inverted T-waves in V1-V6 and inferior leads (II, III, aVF). Reproduced with permission from te Rijdt et al. (2019) [47].

**Figure 17:**
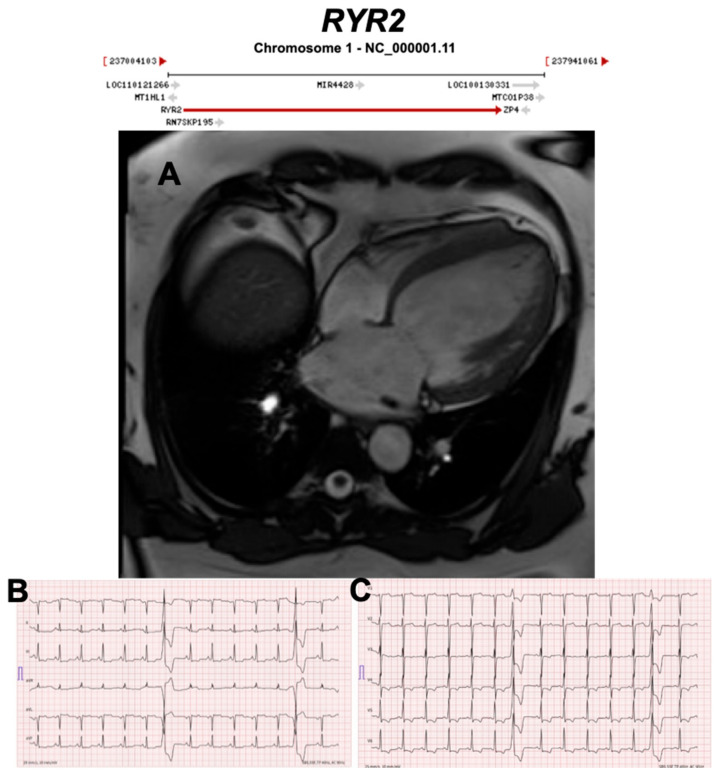
Exemplar MRI and ECG finding found in ACM patients with *RYR2* mutations. (**A**) CMR of a patient with RYR2 p.Trp98Ter mutation showing dilated cardiomyopathy; (**B**,**C**) 12-lead ECG showing inverted T waves in leads II, III, aVF, V3–V6, and two premature ventricular complexes originating from the anterobasal left ventricle. Reproduced with permission from Costa et al. (2020) [48].

**Figure 18:**
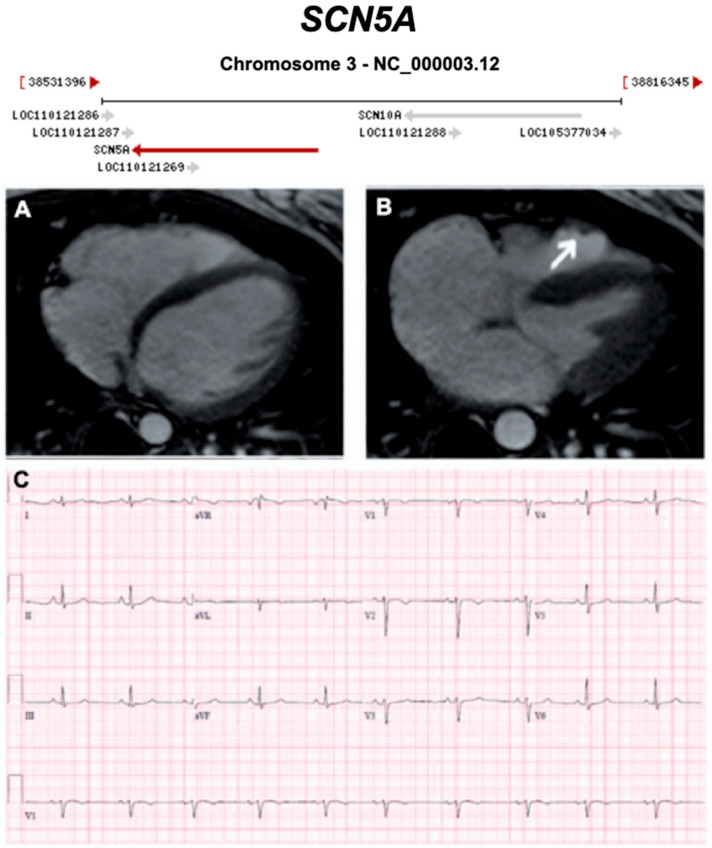
Exemplar MRI and ECG finding found in ACM patients with *SCN5A* mutations. (**A**,**B**) CMR of a female patient with *SCN5A* mutation (p.Arg1898His) showing end-diastolic and end-systolic state, respectively. The white arrow shows an enlarged right ventricle and dyskinesia in the RV outflow tract; (**C**) a 12-lead ECG of the same patient showed T-wave inversion in V1–2. Reproduced with permission from te Riele et al. (2017) [49].

**Figure 19:**
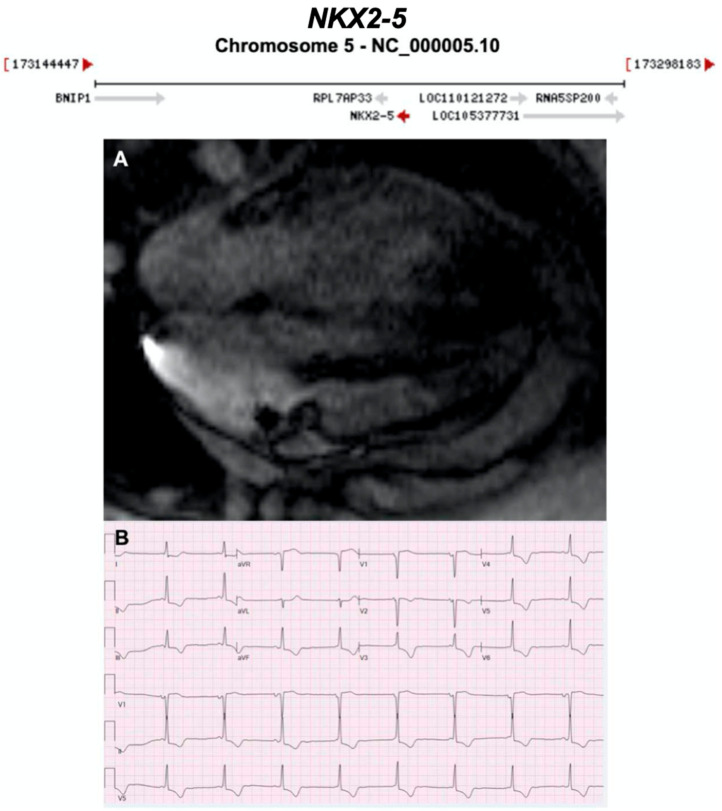
Exemplar MRI and ECG finding found in ACM patients with *NKX2-5* mutations. (**A**) A cardiac MRI of a patient with *NKX2-5* c.471_472delCA variant showing non-dilated hypokinetic ventricle, no valvular heart disease, no LGE; (**B**) ECG finding of a different patient with the same *NKX2-5* mutation as in (**A**). CMR = cardiovascular magnetic resonance; ECG = electrocardiogram; LGE = late gadolinium enhancement; LV = left Ventricle; RV = right Ventricle. Gene location images taken from https://www.ncbi.nlm.nih.gov/gene/.

**Table 1:** Summary of evolving definition and classification of cardiomyopathies and ACM.

**1957**	**The Term ‘Cardiomyopathy’ First Introduced** [13]
	Bridgen describes cardiomyopathies as ‘uncommon, noncoronary myocardial diseases’ and describes some general features found.
**1980**	**WHO-ISFC Task Force Report on Definition and Classification of Cardiomyopathies** [14]
	Cardiomyopathies defined as ‘heart muscle diseases of unknown cause’. Classified into four morphological phenotypes: DCM, HCM, RCM, and Unclassified cardiomyopathy.
**1977**	**ARVD First Recognised and Described** [15]
	Fontaine et al. coin and first recognise ARVD when mapping and treating VT originating from the RV. They propose this new disease be termed ‘dysplasia’ due to the likely theory of a postnatal developmental disorder.
**1982**	**ARVD First Comprehensive Classical Description** [3]
	Marcus et al. comprehensively describe ARVD in a case series of 24 patients.
**1988**	**ARVC First Described** [16]
	Thiene et al. describe ARVC in group of young adults who died from SCD mostly during exercise.
**1994**	**ESC-ISFC Task Force Diagnostic Criteria for ARVD/C** [17]
	Proposed new criteria for the clinical diagnosis of ARVD/C using structural, histological, electrocardiographic, arrhythmic, and familial features.
**1995**	**Updated WHO-ISFC Task Force Report on Definition and Classification of Cardiomyopathies** [18]
	Cardiomyopathies re-defined as ‘diseases of the myocardium associated with cardiac dysfunction’—broadening the definition to include known causes of myocardial disease. Classification split into two groups: primary (intrinsic to myocardium) and specific (secondary to external processes). Fifth phenotype added to primary group: ARVC.
**2006**	**AHA Statement on Contemporary Definitions and Classifications of the Cardiomyopathies** [19]
	The AHA propose a new aetiological-based categorisation split into primary (involving only the heart) and secondary (generalised multiorgan involvement) cardiomyopathies. These are subdivided into genetic, mixed, and acquired forms—importantly genetic forms are given their own designation for the first time. Ion channel disease added to the classification.
**2008**	ESC Working Group on Myocardial and Pericardial Diseases: Classification of the Cardiomyopathies: A Position Statement * [20]
	Cardiomyopathies defined as ‘A myocardial disorder in which the heart muscle is structurally and functionally abnormal, in the absence of coronary artery disease, hypertension, valvular disease and congenital heart disease sufficient to cause the observed myocardial abnormality.’ ESC divides cardiomyopathies into five known morphological phenotypes: (1) DCM, (2) HCM, (3) RCM, (4) ARVC, and (5) unclassified cardiomyopathies, which are further categorised into familial and nonfamilial groups.
**2010**	**Revised Task Force Diagnostic Criteria for ARVC** [5]
	Proposed an updated diagnostic scheme for ARVC based on (1) functional and structural characterization of the right ventricle, (2) histopathological characterisation, (3) repolarisation, (4) depolarisation abnormalities, (5) arrhythmias, and (6) family history and genotype.
**2013**	**MOGE**(**S**) **Classification of Cardiomyopathies** [21]
	Proposed a descriptive genotype-phenotype nosology system based on five attributes: **M**orphofunctional characteristics, **O**rgan involvement, **G**enetic/familial inheritance pattern, A**E**tiological annotation, Functional **S**tatus of patients.
**2019**	**2019 Definition and Treatment of Arrhythmogenic Cardiomyopathy: an Updated Expert Panel Report** [22]
	A consensus statement compiled following a moderated roundtable discussion of an international group of experts in 2017, Athens, Greece, which defined arrhythmogenic cardiomyopathy as a ‘family of diseases that feature structural myocardial abnormalities (identified by macro- and microscopic pathological examination besides cardiac imaging) and ventricular arrhythmia’. Terms include ARVC, ALVC, aDCM, isolated non-ischaemic scar and hypokinetic, non-dilated left ventricle. Fundamental aspects include arrhythmia, electrical abnormalities, structural abnormalities (important not essential chamber dimensions or contractility but tissue characterisation is important), heritability (family history, genetic aetiology, cardiocutaneous, neuromuscular features), exclusion of phenocopies (such as sarcoidosis, myocarditis, PHTN, and congenital abnormalities).
**2019**	**2019 HRS Expert Consensus Statement On evaluation, Risk Stratification, and Management of Arrhythmogenic Cardiomyopathy** [1]
	New definition and classification proposed for ARVC (now referred to as ACM) defined as an ‘arrhythmogenic heart muscle disorder not explained by ischemic, hypertensive, or valvular heart disease.’ This is broad and include classical ARVC, ALVC, arrhythmogenic biventricular cardiomyopathy, as well as other cardiomyopathies such as amyloidosis, Chagas, sarcoidosis, myocarditis, HCM.
**2020**	**Diagnosis of Arrhythmogenic Cardiomyopathy: The Padua Criteria** [23]
	These criteria build on the 2010 TFC multi-parametric approach to include biventricular and ALVC involvement. Of note, introduction of new LV ECG criteria and tissue characterisation by CMR.

* Most widely used classification of cardiomyopathies currently. ACM = arrhythmogenic cardiomyopathy; aDCM = arrhythmogenic dilated cardiomyopathy; AHA = American Heart Association; ALVC = arrhythmogenic left ventricular cardiomyopathy; ARVC = arrhythmogenic right ventricular cardiomyopathy; ARVD = arrhythmogenic right ventricular dysplasia; CMR = cardiac MRI; DCM = dilated cardiomyopathy; ECG = electrocardiogram; ESC = European Society of Cardiology; HCM = hypertrophic cardiomyopathy; ISFC = International Society and Federation of Cardiology; LV = left ventricle; RCM = restricted cardiomyopathy; PHTN = pulmonary hypertension; TFC = Task Force Criteria; WHO = World Health Organisation.

**Table 2:** Summary table for the genes implicated in ACM

Gene	Protein Type	Frequency *	Predominant Inheritance Pattern	Predominant Ventricular Disease **	OMIM Entry	Exon Location; Exon Count	Remarks	Gene-Disease Validity Classification ***
*PKP2*	Desmosome	20–45%	AD	RV, BIV	ARVC9	12p11.21; 14	Classical ARVC, AR also reported	Definitive for ARVC
*DSG2*	Desmosome	4–15%	AD	RV, LV, BIV	ARVC10	18q12.1; 16	Frequent LV involvement, AR also reported	Definitive for ARVC
*DSP*	Desmosome	1–13%	AD	LV, BIV	ARVC8	6p24.3; 24	Cardiocutaneous Syndrome AR (Carvajal), can also have cardiocutaneous with AD	Definitive for ARVC
*DSC2*	Desmosome	1–7%	AR	RV, BIV	ARVC11	18q12.1; 18	Cardiocutaneous AR	Definitive for ARVC
*JUP*	Desmosome	0–1%	AD and AR	RV, BIV	ARVC12	17q21.2; 19	Cardiocutaneous syndrome AR (Naxos)	Definitive for ARVC
*TTN*	Sarcomere	18%	AD	RV, LV, BIV	-	2q31.2; 365	DCM	Limited for ARVC
*LMNA*	Nuclear Intermediate Filament	3–4%	AD	LV, BiV	-	1q22; 17	DCM, Lipodystrophies, Myopathies	Limited for ARVC
*DES*	Cytoplasmic Intermediate Filament	<1%	AD	LV, BIV	ARVC7	2q35; 9	Myofibrillar myopathy, DCM	Moderate for ARVC
*FLNC*	Actin cross-link	0–3%	AD	LV, BIV	-	7q32.1; 48	High propensity for arrhythmia, SCD, structural abnormalities, LGE and HF. Should consider ICD as a primary prevention.	-
*TJP1*	Intercalated Disc	0–4% [31]	AD		-	15q13.1; 33		Limited for ARVC
*CDH2*	Intercalated Disc	0–2%	AD	RV, BIV	-	18q12.1; 19	-	Limited for ARVC
*CTNNA3*	Intercalated Disc	<1%	AD	RV, BIV	ARVC13	10q21.3; 27	Low penetrance	Limited for ARVC
*TMEM43*	Nuclear Envelope	<1%	AD	RV, BIV	ARVC5	3p25.1; 13	Founder variant in Newfoundland, SCD	Definitive for ARVD 5
*PLN*	Calcium Regulation	0–12%	AD	LV, BIV	-	6q22.31; 2	Founder mutation in Netherlands. High risk of SCD—may consider ICD as primary prevention	Definitive for CMP
*RYR2*	Calcium Regulation	9%	AD	Exon 3 deletion DCM	ARVC2	1q43; 107	CPVT	Refuted for ARVC, Definitive for CPVT
							Conflicting evidence of misdiagnosis CPVT as ARVC with the exception of exon 3 deletions associated with structural abnormalities.	
*SCN5A*	Sodium Channel	0–2%	AD	LV, BIV	-	3p22.2; 29	BrS, LQTS Type 3, AF	Limited for ARVC, Definitive for BrS
*TGFB3*	Cytokine	Unknown	AD	RV	ARVC1	14q24.3; 8	-	Limited for ARVC
*RBM20*	Splicing Factor	Unknown	AD	LV	-	10q25.2; 16	High risk of SCD—considered primary prevention by ICD	Definitive for DCM
*BAG3*	Chaperone	Unknown	AD	LV	-	10q26.11; 4		-
*NKX2-5*	Homeobox	Unknown	AD		-	5q35.1; 3		-

(Modified from Gandibakhch et al. (2018)). * Taken from Gandibakhch et al. (2018) [32] unless specified. ** Taken from Elliott et al. (2019) [22]. *** Taken from ClinGen [33]. AD = autosomal dominant; AF = atrial fibrillation; AR = autosomal recessive; ARVC = arrhythmogenic right ventricular cardiomyopathy; BIV = biventricular disease; BrS = Brugada syndrome; CPVT = catecholaminergic polymorphic ventricular tachycardia; DCM = dilated cardiomyopathy; HF = heart failure; LGE = late gadolinium enhancement; LQTS = long QT Syndrome; LV = left ventricle; RV = right ventricle; SCD = sudden cardiac death.

**Table 3:** Future directions: research priorities in ACM.

Epidemiology and screening of ACM	Highly variable
	Under-studied, underdiagnosed
	GWAS may be helpful to establish associations between patients’ genotype and phenotype; and to test existing genetic risk scores from other cardiac phenotypes with ACM phenotypes for associations. Moreover, this could be potentially developed into a screening test
Clinical heterogeneity	Inter and intra-familial variability (even with some mutations)
	Deep phenotyping with bipolar and unipolar voltage maps of RV, RVOT, LV, and with phenocopies
Mechanisms of ACM	Pathophysiology and disease progression
	Myocarditis as a possible trigger and predisposition to arrhythmias
	Animal models may be used to study the effect of protein mutations on ACM phenotypes, e.g., titin mutation and its link to heart failure
Genetics	40% undefined genetically
	Genotype-phenotype correlation
	More precise definition of clinical phenotype
	GWAS and phenotype-genotype for effect modifiers?
Desmosome	Study extra-cardiac desmosome expression such as buccal cells and skin
Inflammation and myocarditis	Investigate role of inflammation in animals and humans
	Discern if ACM patients more susceptible to myocarditis
	Is inflammation primary or secondary?
	Is myocarditis a trigger for arrhythmogenicity?
	Is myocarditis an acute phase of ACM?
Predicting SCD risk	Refine the criteria for ICD implantation in ACM patients e.g., balancing the benefits and risks of ICD, specifying the type of ICD (TV vs. SC)
	Validation Hopkins Primary Prevention ARVC SCD risk calculator
	Evaluating blood biomarkers longitudinal studies
	Systematic follow-up of G+/P+, G+/P+, G−/P− understand evolution of arrhythmia
	Use of non-ICD therapies to manage arrhythmia risk
Other therapies	Trials using standard anti-HF regimens in ACM (β-blockers, Angiotensin-converting enzyme, Angiotensin II receptor blocker, Mineralocorticoid receptor antagonist, Angiotensin receptor neprilysin inhibitor)
	Disease-specific pathways to identify novel targets
	Skin and hair for testing novel therapies
	Precision medicine with the use of ‘avatars’ for testing therapies
	Establish trials with surrogate endpoints to expedite drug delivery

GWAS = genome-wide association studies; G+ = genotype positive, G− = genotype negative, P+ = phenotype positive, P− = phenotype negative, HF = heart failure; RV = right ventricle; RVOT = right ventricular outflow tract; SC = subcutaneous; SCD = sudden cardiac death; TV = transvenous.

**Table 4:** Functional Characterisation of Variants.

DNA level	Genetic interaction mapping
	DNA/protein interactions
	DNA accessibility assays
RNA level	Microarrays
	SAGE (Serial Analysis of Gene Expression)
	RNA sequencing
	MPRAs (Massive Parallel Reporter Assays)
	STARR-sequencing (similar to MPRA)
	Perturb-sequencing (couples CRISPR with gene knockdown single gene expression)
Protein level	Yeast two-hybrid systems (bait with prey originally based on transcription factor GAL4)
	AP/MS (Affinity Purification and Mass Spectrometry)
	Deep mutational scanning
Loss of functional techniques	Mutagenesis (deletional or insertional mutagenesis laborious pre-CRISPR)
	RNAi (RNA interference using s20 base dbRNA transfection using siRNA or virally-delivered short hairpin RNAs
	CRISPR
Functional annotation of genes	Genomic annotation
	Rosetta Stone approach [150]
Bioinformatics	Clustering
	Principal component analysis
	Machine learning
	Artificial neural networks
	Support vector machines (supervised machine learning)
	Gene ontology (DAVID)
	Gene set enrichment analysis (GSEA)
	Pathway analysis—Ingenuity
	Pathway Studio—Ariadne Genomics
Consortia	ENCODE (Encyclopaedia of DNA Elements)
	GTEx (Genotype-Tissue expression project)

CRISPR = clustered regularly interspaced short palindromic repeats.

## Abbreviations

ACM	Arrhythmogenic Cardiomyopathy
ACMG-AMP	American College of Medical Genetics and Genomics and the Association for Molecular Pathology
aDCM	Arrhythmogenic Dilated Cardiomyopathy
AD	Autosomal dominant
AF	Atrial Fibrillation
AHA	American Heart Association
AJ	Adherens Junction
ALVC	Arrhythmogenic Left Ventricular Cardiomyopathy
AP/MS	Affinity Purification and Mass Spectrometry
AR	Autosomal recessive
ARVC	Arrhythmogenic Right Ventricular Cardiomyopathy
ARVD	Arrhythmogenic Right Ventricular Dysplasia
ASD	Atrial Septal Defect
BiV	Biventricular
BivACM	Biventricular Arrhythmogenic Cardiomyopathy
BrS	Brugada syndrome
CMP	Cardiomyopathy
CMR	Cardiovascular magnetic resonance
CPVT	Catecholaminergic Polymorphic Ventricular Tachycardia
CRISPR	Clustered regularly interspaced short palindromic repeats
DAD	Delayed After Depolarisations
DCM	Dilated Cardiomyopathy
ECG	Electrocardiogram
ENCODE	Encyclopedia of DNA Elements
ESC	European Society of Cardiology
FDG-PET	^18^F-fluorodeoxyglucose positron emission tomography
G+	Genotype negative
G-	Genotype negative
GSEA	Gene set enrichment analysis
GTEx	Genotype-Tissue expression project
GWAS	Genome-wide association studies
HCM	Hypertrophic Cardiomyopathy
HF	Heart failure
hiPSC-CMs	Human induced pluripotent stem cell cardiomyocytes
HNDC	Hypokinetic Non-dilated Cardiomyopathy
HRS	Heart Rhythm Society
ICD	Implantable Cardiac Defibrillator
ID	Intercalated Disc
ISFC	International Society and Federation of Cardiology
LBBB	Left Bundle Branch Block
LGE	Late gadolinium enhancement
LQTS	Long QT syndrome
LV	Left ventricle
LVEF	Left ventricular ejection fraction
MPRAs	Massive Parallel Reporter Assays
MR	Mitral Regurgitation
NSVT	Non-Sustained Ventricular Tachycardia
P+	Phenotype positive
P-	Phenotype negative
PHTN	Pulmonary Hypertension
PVC	Premature Ventricular Contraction
RCM	Restrictive Cardiomyopathy
RV	Right ventricle
RVOT	Right ventricular outflow tract
RWMA	Regional wall motion abnormality
SAGE	Serial Analysis of Gene Expression
SC	Subcutaneous
SCD	Sudden Cardiac Death
SERCA	Sarcoplasmic reticulum Ca^2+^-ATPase
SR	Sarcoplasmic reticulum
TFC	Task Force Criteria
TV	Transvenous
TWI	T Wave Inversion
UTR	Untranslated regions
VT	Ventricular Tachycardia
VTA	Ventricular Tachyarrhythmia
WES	Whole exome sequencing
WGS	Whole genome sequencing
WHO	World Health Organisation
